# Glutamine synthetase (GS) knockout (KO) using CRISPR/Cpf1 diversely enhances selection efficiency of CHO cells expressing therapeutic antibodies

**DOI:** 10.1038/s41598-023-37288-6

**Published:** 2023-06-28

**Authors:** Witsanu Srila, Martina Baumann, Markus Riedl, Kuntalee Rangnoi, Nicole Borth, Montarop Yamabhai

**Affiliations:** 1 Molecular Biotechnology Laboratory, School of Biotechnology, Institute of Agricultural Technology, Suranaree University of Technology, Nakhon Ratchasima, Thailand; 2 Austrian Centre of Industrial Biotechnology (ACIB), Vienna, Austria; 3 Department of Biotechnology, University of Natural Resources and Life Sciences (BOKU), Vienna, Austria

**Keywords:** Biological techniques, Biotechnology, Molecular biology

## Abstract

The glutamine synthetase (GS)-based Chinese hamster ovary (CHO) selection system is an attractive approach to efficiently identify suitable clones in the cell line generation process for biologics manufacture, for which GS-knockout (GS-KO) CHO cell lines are commonly used. Since genome analysis indicated that there are two GS genes in CHO cells, deleting only 1 GS gene could potentially result in the activation of other GS genes, consequently reducing the selection efficiency. Therefore, in this study, both GS genes identified on chromosome 5 (GS5) and 1 (GS1) of CHO-S and CHO-K1, were deleted using CRISPR/Cpf1. Both single and double GS-KO CHO-S and K1 showed robust glutamine-dependent growth. Next, the engineered CHO cells were tested for their efficiency of selection of stable producers of two therapeutic antibodies. Analysis of pool cultures and subclones after a single round of 25 µM methionine sulfoxinime (MSX) selection indicated that for CHO-K1 the double GS5,1-KO was more efficient as in the case of a single GS5-KO the GS1 gene was upregulated. In CHO-S, on the other hand, with an autologously lower level of expression of both variants of GS, a single GS5-KO was more robust and already enabled selection of high producers. In conclusion, CRISPR/Cpf1 can be efficiently used to knock out GS genes from CHO cells. The study also indicates that for the generation of host cell lines for efficient selection, the initial characterisation of expression levels of the target gene as well as the identification of potential escape mechanisms is important.

## Introduction

The growing significance of biologics has resulted in the development of technologies for high quality and productivity in different types of expression systems^1^. Among mammalian cell lines, Chinese hamster ovary (CHO) cells are the key platform for biopharmaceutical production because they can secrete recombinant antibodies (rAbs) at relatively high levels and provide a human-like glycosylation pattern with little concern for immunogenic modifications^2^. Moreover, during the last years their genomes have been extensively studied, facilitating genetic manipulation^3–7^. Manufacturing processes for biologics have been improved significantly with respect to final yield over the past decades with titers up to or higher than 10 g/L^8,9^.

Establishing a stable cell line is still a critical step for therapeutic Ab manufacturing. This procedure includes several rounds of selection and amplification, followed by isolation, screening, and characterization of a large number of clones^10,11^. In general, a cell line development process (CLD) takes approximately 5–12 months because the variation in phenotypes of subclones is high so that many have to be screened to identify those few clones that perform at sufficiently high level for industrial manufacturing. During CLD, the chance that the gene of interest (GOI) will be integrated into a highly transcribed or hot spot region for the cells to produce adequate levels of rAb is extremely low^12,13^. Since clonal variation is high, cell line development is both time-consuming and laborious^14,15^. Therefore, for the biologics industry, it is essential to improve the efficiency of selection of cells with high expression level, so as to reduce CLD timelines and/or to increase yield, which will eventually reduce manufacturing costs for the pharmaceutical industry, especially for lower to middle income countries.

Two main systems i.e., dihydrofolate reductase (DHFR)-based methotrexate (MTX) and glutamine synthetase (GS)-based methionine sulfoximine (MSX) selection systems, have been used to select stable Ab producing clones. The DHFR system is the standard method to accomplish the production of recombinant therapeutic proteins despite its extensive stepwise gene amplification procedure which makes it laborious and time-consuming. The GS selection system which is based on the reduction of GS activity using MSX as inhibitor in combination with GS gene deletion is more efficient as it requires a shorter timeline to generate stable cell lines^16,17^. Therefore, the GS-CHO system is an attractive approach to efficiently identify high producing and stable clones^18^. Recent expiration of the patent for the GS-based system has led to an interest in further improvements. Previous studies have shown that reduction of GS activity using an attenuated SV40E promoter for GS geneexpression^17^ or utilization of attenuated GS^15^, could lead to more efficient cell line generation in the absence of MSX, simply by selection in glutamine free media.

Several techniques have been used to delete the GS gene, such as zine finger nuclease (ZEN)^19^ and transcription factor-like effector nuclease (TALENs)^16^. In this study, to further improve the selection stringency and assure long-term stability of CHO cells, an advanced CRISPR technology was used to engineer 2 CHO cell lines, i.e., CHO-K1 and S^4^. The hypothesis is based on the fact that bioinformatic analyses of the CHO genome indicated that there are more than one GS genes in the CHO cells, albeit not necessarily expressed under normal conditions or to the same level. Deleting the main GS gene may lead to the activation of the second copy, if the latter is normally not highly expressed or not expressed at all, resulting in a reduction in selection stringency by MSX. In this report, both the highly expressed GS gene on chromosome 5 (GS5) and the lowly expressed GS on chromosome 1 (GS1), were deleted in CHO-K1 and S by CRISPR/Cpf1. Successful GS-deletion of single (GS5) and double (GS5 and GS1) engineered CHO-K1 and S were confirmed by deletion and non-deletion, RT-quantitative PCR prior to testing for their efficiency in the selection of stable production cell lines for two therapeutic Abs, i.e., Adalimumab and Trastuzumab.

## Results and discussion

### Identification and expression of GS genes in CHO cells

**Figure 1:**
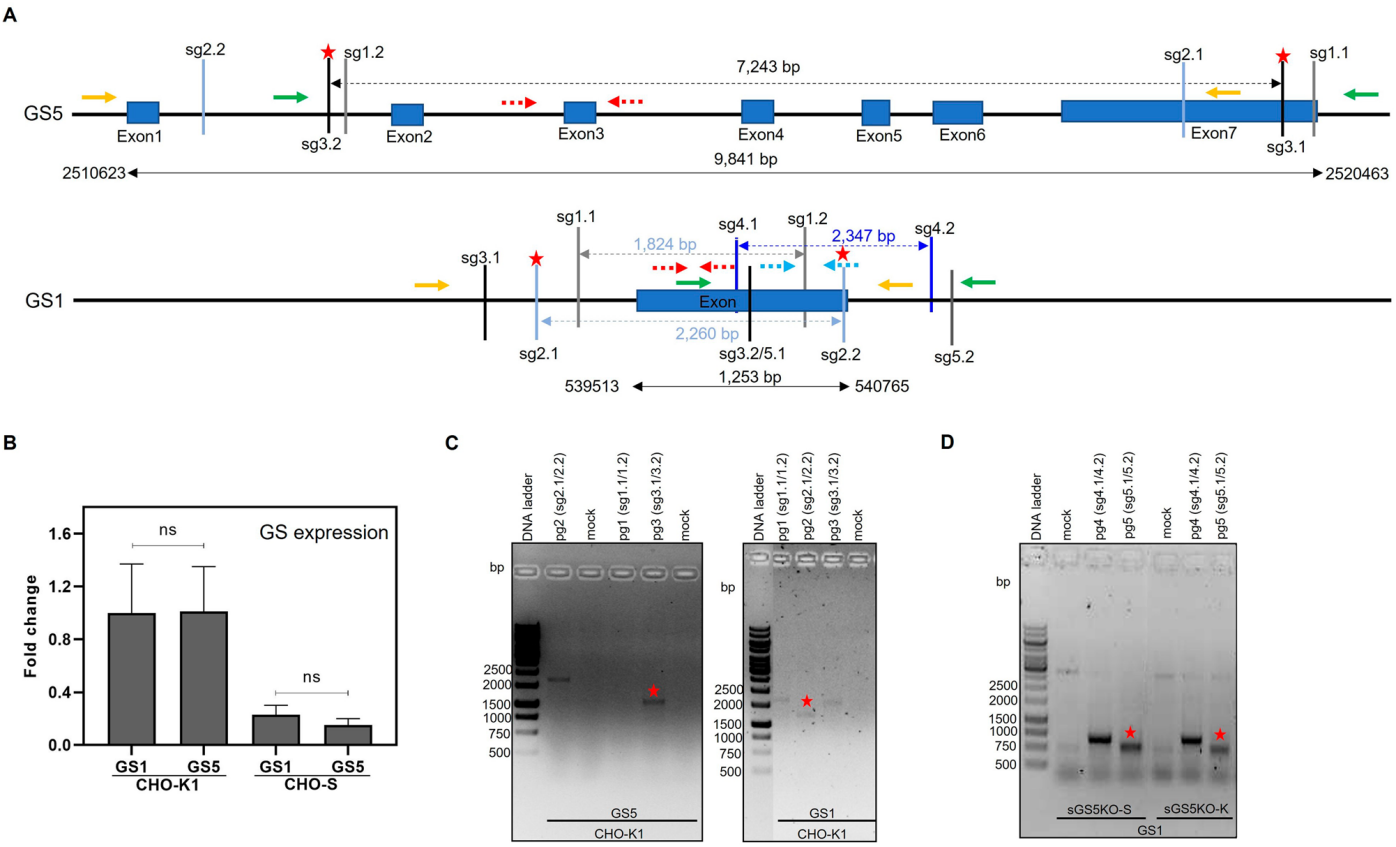
The endogenous GS genes in CHO-K1 genome database and possible pgRNA for knockout the GS genes. (**A**) Schematic representation of GS genes in CHO-K1 genome and sgRNA positions. The sgRNA positions are shown as vertical lines. The solid arrows indicate primers to detect the deletion PCR product and dashed arrows indicate primers to detect the non-deletion PCR product. Color of arrows represent each pair of PCR reaction. (**B**) Expression of various GS genes in CHO-K1 and S. Relative mRNA expression of two GS genes in CHO-K1 and S, normalized to GAPDH, are shown. The data represent mean ± SD from a quadruplicate representative example. Statistical analysis was performed with the GraphPad Prism 8.0.1 software using Student’s *t*-test and followed by Welch’s test. Symbol “^a^, ^b^, ^c^” indicate a statistically significant difference *p* < 0.001, 0.01 and 0.05, respectively and ns indicates no significant difference. (**C**) Evaluation of pgRNA efficiency for GS gene deletions. Each vector carrying pgRNA was transfected into CHO-K1. After transfection, the transfected cells were tested for gene deletion by PCR. The pool cells showing the deletion amplicon indicated that the designed sgRNA pairs can be used to create GS-KO CHO cells. The illustrated gel is the representative example of one biological replicate. (**D**) Assessment of pgRNA efficiency for the deletion of GS1 gene in sGS5KO-K and sGS5KO-S cells by deletion PCR. The original gels are presented in Supplementary Fig. S1).

Database analysis (see Supplementary information 1 indicated that the endogenous GS genes in CHO-K1 include two genes on chromosome 5 (GS5) and chromosome 1 (GS1), for which in-house RNAseq data and previous publication, listed in Supplementary Table S1, showed high and low expression, respectively. The highly expressed GS5 gene is composed of 7 exons, exon 6 carrying the essential sequence for GS activity^20^, while the lowly expressed GS1 has only one exon (Fig. 1A). The GS1 sequence and its expression has been previously verified by PCR amplification of the mRNA^21^. Even if the sequence on chromosome 1 does not consist of the entire GS sequence found on chromosome 5 as depicted in Fig. 1A, it contains the substrate binding, termed beta-grasp domain with the molecular function of “Glutamate-ammonia-ligase activity” as described in https://www.ebi.ac.uk/interpro/entry/InterPro/IPR008147/. Thus, at least residual function of glutamine synthesis can be inferred. The expression level of these genes was confirmed by the relative expression levels of mRNA by RT-qPCR with specific primers (Supplementary Table S2) for both CHO-K1 and S as shown in Fig. 1B. The results indicated that both GS5 and GS1 genes were expressed at a comparable level in both CHO-K1 and S. Notably, the expression level of both GS1 and GS5 genes in CHO-K1 are much higher than those in CHO-S (Fig. 1B). These findings were different from the mapping of RNAseq transcripts had suggested, which could be because the program for predicting the expression of transcripts was not able to properly assign reads from the two variants due to sequence homologies. Verification of the true expression of the gene from each chromosome would require long read sequencing of mRNA and identification of the translated protein, which, again in view of the sequence homology, would be difficult. For the present study it was deemed to be the most straightforward strategy to delete both genes using a paired guide RNA(pgRNA)/CRISPR-Cpf1 deletion approach^22^.

The main reason for using CRISPR/Cpf1 instead of Cas9 was because this system has already been well established in our laboratory. In other contexts, there are several points which might make it preferable to use CRISPR/Cpf1 rather than Cas9. These include (i) Cpf1 possesses both RNase and DNase activity, which would allow to process the pre-crRNA pairs for use in a pgRNA library approach^23^. The gRNAs are also smaller than those for Cas9 which enables the direct synthesis of pgRNAs for said libraries^24^. (ii) Cpf1's PAM is TTTN. This is particularly useful to edit in AT-rich genomes or regions and multiplexed gene targeting^25,26^. Thus, when it is not possible to identify a suitable PAM sequence for Cas9 in a specific genomic region, Cpf1 is a good alternative.

### Design and assessment of paired guideRNAs (pgRNAs)

Since we hypothesized that single GS deletion might lead to the activation or upregulation of the other GS gene, compromising selection stringency, we decided to delete both endogenous GS genes in CHO-K1 and S cells. To do this, three different pgRNAs (pg1 (sg1.2/2.2), pg2 (sg2.1/2.2) and pg3 (sg3.1/3.2)) for each GS gene were designed (Fig. 1A), cloned into pY010 (AsCpf1), and transfected into CHO-K1 cells to identify suitable pgRNAs for the deletion of the GS genes. The efficiency of pgRNAs was evaluated by deletion PCR as described in the method section. The position of PCR primers used in this study are indicated in Fig. 1A. Different colors of the solid arrow represent different primer pairs for deletion PCR; while different colors of the dotted arrows indicated primer pairs for non-deletion PCR. The visible band of PCR products as shown in Fig. 1C indicates that genes were successfully deleted. From these results, pairs for GS5 (pg3:sg3.1/3.2) and GS1 (pg2:sg2.1/2.2), which showed the strongest deletion PCR band intensity and covered the biggest genomic deletions (marked with asterisk in Fig. 1A and C) were selected for the engineering of GS-KO CHO-K1 and S cell lines in the next step.

### Engineering of GS-KO CHO cells by CRISPR/Cpf1

**Figure 2:**
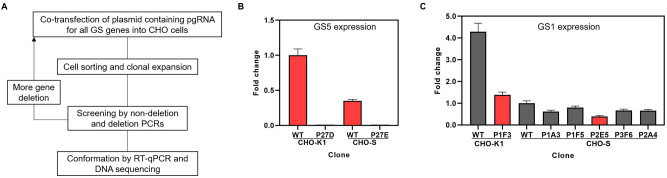
Generation of GS-KO CHO-K1 and S cells. (**A**) An overview of the process for the construction of multi-GS-KO CHO-K1 and S cells. (**B**) the GS5 expression level of CHO-K1 P27D (sGS5KO-K) and CHO-S P27E (sGS5KO-S) clones, relative to GAPDH expression by RT-qPCR. (**C**) The GS1 expression level in candidate clones determined by RT-qPCR, relative to GAPDH expression. WT; wild type.

A summary of the workflow for the generation of GS-KO CHO cell lines in this study is shown in Fig. 2A. CHO cells were co-transfected with appropriate pairs of gRNAs and subcloned by fluorescence-activated cell sorting (FACS). Clone screening was done by non-deletion PCR followed by screening for deletion PCR of various GS-KO. After obtaining confirmed GS-KO cell lines, a second round of cell engineering was performed by transfection with new pairs of gRNAs, targeting the next GS region to be deleted. The deletion of GS genes was subsequently confirmed by RT-qPCR and DNA sequencing.

### Results from the first round of transfection using 2 pgRNAs

The first attempt to generate GS-KO CHO-K1 and S were performed using the two selected pgRNA combinations together as described above. FACS resulted in a total of 144 (for CHO-S) and 96 (for CHO-K1) clones. These clones were first evaluated by non-deletion PCR for the absence of GS5 gene because it was annotated as the most highly expressed gene. Among these, 40 clones of CHO-S and 8 clones of CHO-K1 showed the absence of GS5 non-deletion amplicon. After that, non-deletion PCR to check for KO of GS1 was sequentially performed. Unfortunately, we were not able to obtain any clones that had a confirmed GS1 deletion. Therefore, from our first attempts to delete both GS genes, apparently only the KO of highly expressed GS5 gene was successful. Subsequently, biallelic deletion of the GS5 gene from the selected clones were confirmed by deletion PCR (Supplementary Fig. S2), and our results indicated that only one clone with full deletion of both GS5 alleles each was obtained for CHO-K1 (P27D) and S (P27E). These clones were designated as sGS5KO-K and sGS5KO-S, respectively. These results are not unexpected as it has been previously reported that gene deletion by CRISPR system frequently resulted in either indel formation at the sgRNA recognition site or monoallelic deletion^27^.

Finally, RT-qPCR was performed to validate the complete KO of GS5 expression as demonstrated in Fig. 2B. Sanger sequencing of the deletion PCR product when using genomic DNA (gDNA) as a template was performed to identify the precise CRISPR-engineered site. Sequencing results of sGS5KO-S and sGS5KO-K clones (Supplementary Fig. S3A) confirmed biallelic deletion with minor differences in the deleted regions.

In summary, our first attempt to create double GS-KO cells resulted in only one sGS5-KO clone for each CHO cell type.

### Second round of GS-KO using new pgRNAs

To further delete the GS1 gene from single GS5-KO cells obtained from the previous step, the efficiency of pgRNAs was re-assessed again for sGS5KO-S and sGS5KO-K cells as shown in Supplementary Fig. S4. However, after transfection of the effective pgRNAs and screening for GS1-KO by non-deletion and deletion PCRs, no successful biallelic deletion of GS1 was achieved. These results could be the result of transfected pgRNAs for two genes in the first round, where indels were generated that interfere with the deletion in the second round. Another possibility is that the pgRNAs cause deletion of an essential region. Therefore, in the second attempt to generate GS-KO cell engineering, new pgRNAs for GS1 (pg4:sg4.1/4.2 and 5:sg5.1/5.2), specific to internal and downstream (long noncoding (lnc) transcript) of the GS1 gene, were designed as depicted in Fig. 1A. The efficiency of the new pgRNAs was evaluated by deletion PCR as shown in Fig. 1D. The pgRNA 5 (pg5:sg5.1/5.2) was selected for the deletion of GS1.After transfection and sorting by FACS, subclones were screened by non-deletion, followed by deletion PCRs as previously described. Five candidates of sGS5KO-S (P1A3, P1F5, P2E5, P3F6 and P2A4) and 1 candidate of sGS5KO-K (P1F3) were obtained (Supplementary Fig.S5).

### Successful generation of double GS-KO CHO cell lines

RT-qPCR was performed to confirm full deletion of the GS1 gene in the 6 double GS-KO clones (Fig. 2C). Among the five CHO-S candidate clones, the expression of GS1 decreased 0.20–0.61 fold, compared to wild-type (WT) CHO-S cells. For the one candidate clone of CHO-K1, the expression of GS1 reduced 0.68 fold, compared to WT CHO-K1 cells. Clone P2E5 from CHO-S and P1F3 from CHO-K1 showed lowest GS1 expression. From these results, clones P1F3 and P2E5 were selected, designated as dGS5,1KO-K and dGS5,1KO-S and used for the next step. Sequencing results of these two double GS-KO CHO clones confirmed that the GS1 gene was deleted (Supplementary Fig. S3B).

Successful generation of single and double GS-KO CHO K-1 and S cells demonstrated the high efficiency of CRISPR/Cpf1 system in complete biallelic deletion of CHO cells. In addition to also deleting the GS1 gene, KO of GS5 gene of the GS-KO cells in this study is different from other GS-KO cells because the entire GS5 gene was deleted by CRISPR technology instead of deletion of only position in exon 5 or 6, which contain the key sequence critical for GS activity by using zinc finger nuclease (ZFN)^19^ or transcription activator-like effector nucleases (TALEN) based-technology^15^. Deletion of the whole gene has an advantage over frameshift mutations which could cause protein misfolding that may not only cause a loss of function but create unknown side effects and stress responses leading to cell impairment with multiple dysfunction^22^.

### Growth characteristics of different GS-KO CHO cells

**Figure 3:**
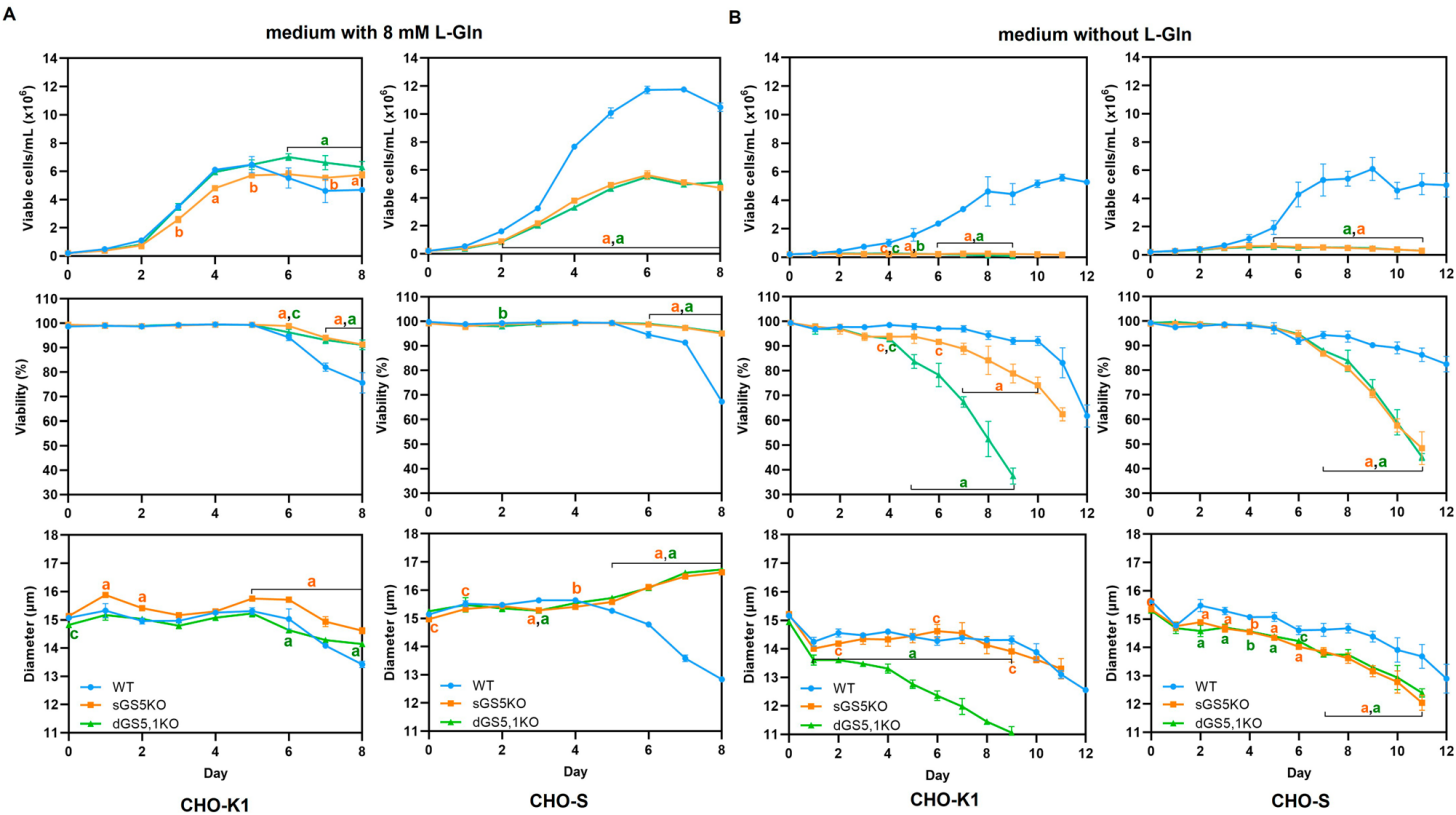
Growth behavior of GS-KO CHO cell lines. (**A**) The cells were cultured in medium supplemented with L-Gln. (**B**) The cells were cultured in medium without L-Gln. All values represent mean ± S.D. of triplicates. Statistical analysis was performed with the GraphPad Prism 8.0.1 software using two-way ANOVA and followed by Dunnett’s multiple comparison test. Symbol “^a^, ^b^, ^c^” indicate a statistically significant difference versus wild type (WT) *p* < 0.001, 0.01 and 0.05, respectively.

The growth behavior of the generating engineered CHO cells was assessed to validate the disruption of GS activity. In case of CHO-K1 cells, the growth characteristic of GS-KO and WT CHO-K1 were similar, except for cell viability, which also dropped after day 5 in the WT. Both single (sGS5KO-S) and double (dGS5,1KO-S) CHO-S clones had equal viable cell density (VCD), viability, and cell diameter in medium containing L-Gln throughout the experiment (Fig. 3A). The growth of these two clones was significantly slower compared with the WT CHO-S cell, which reached a maximum VCD of 11.8 × 10^6^ cells/mL on day 7. Due to this higher cell density and consequently higher substrate utilisation, the WT cells’ viability and size decreased after day 5.

The GS pathway converts glutamate and ammonia into L-Gln. The GS-KO CHO clones that disrupt the activity of endogenous GS enzyme must be grown in media supplemented with L-Gln to survive, where Gln is first used for protein translation, but a significant part of it also is deaminated to enter the TCA as alpha-keto glutaric acid, thus providing a major contribution to energy production, which the GS-KO-CHO lack. This could possibly explain the differences in growth behavior, although previous work has also shown that cells can adapt to growth in Gln free medium without any changes in final cell density or growth behavior^28^. However, in the present study no such adaptation time was granted to the cells.

When the cells were cultured in Gln-free medium, the engineered CHO-K1 and S cells did not grow or survive (Fig. 3B). In contrast, WT CHO-K1 and S cells continued to grow in the medium without L-Gln as they are able to synthesize L-Gln from endogenous GS genes. The viability of GS-KO CHO-K1 and S clones significantly decreased after day 4 (for dGS5,1KO-K) or day 6 (for sGS5KO-K and both dGS5,1 and sGS5 CHO-S), as opposed to their WT CHO cells. All cells revealed a reduction of cell diameter overtime in Gln-free medium. For CHO-K1, the dGS5,1KO-K had a more rapid decline in viability and a smaller cell size than of the single sGS5KO-K. This could be a consequence of the high expression of GS1 in these cells which enabled partial rescue in the absence of glutamine. In CHO-S, the “rescuing” effect of GS1 was smaller, presumably due to the lower expression of both GS variants in this cell line.

### The expression level of GS genes in single and double GS-KO CHO cells

**Figure 4:**
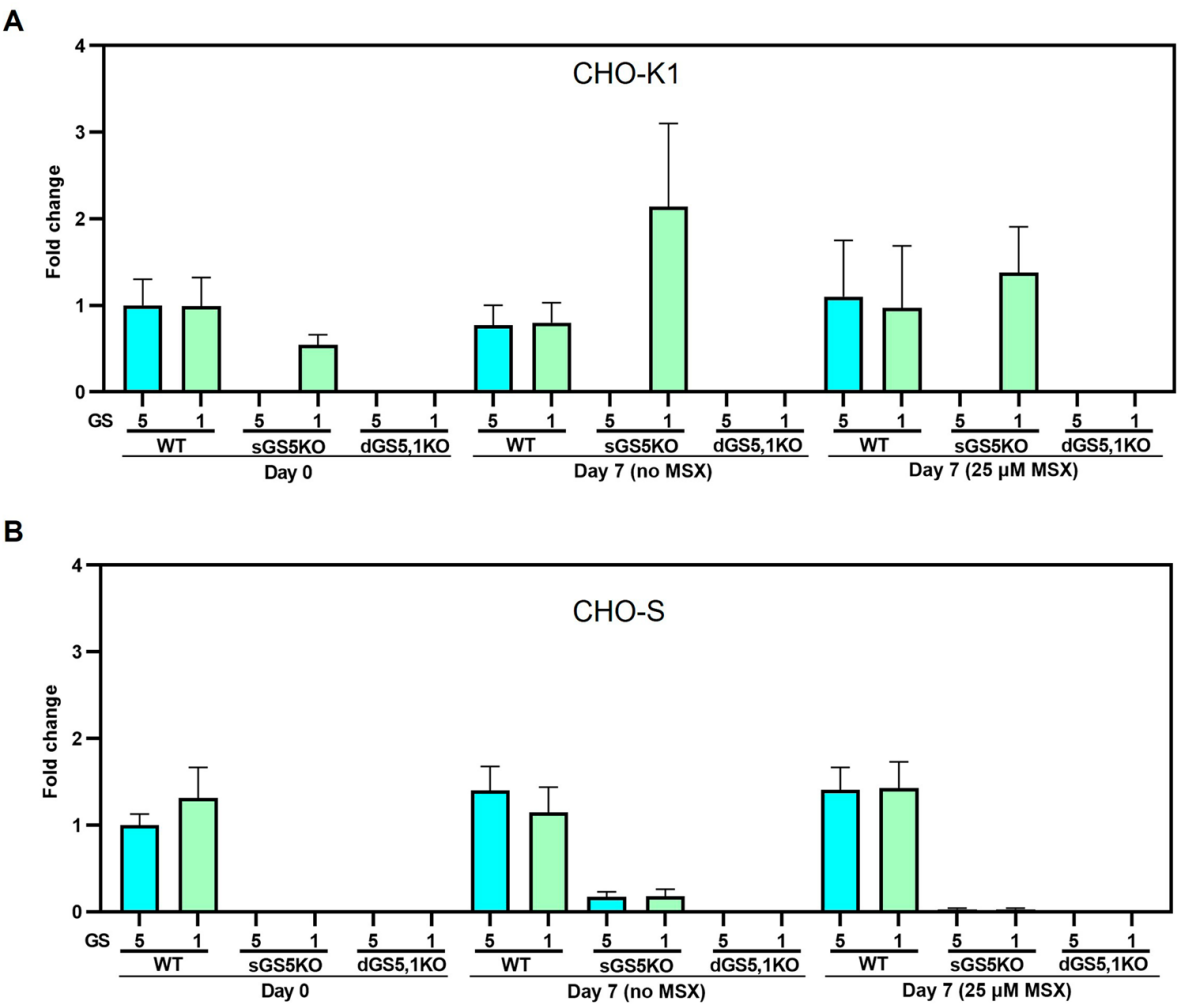
The mRNA expression level of GS genes in each GS-KO clone on day 7 of a batch culture in GS selection medium either with or without MSX.

The mRNA expression levels of GS5 and GS1 genes in selected engineered GS-KO CHO cells were determined on day 7 of batch culture in medium without L-Gln either with or without MSX supplement (Fig. 4). Regardless of the batch culture conditions, both GS5 and GS1 genes were not expressed in double GS-KO CHO clones. For WT CHO-K1 and S, the mRNA expression of both GS1 and 5 were as expected, where the addition of 25 µM MSX didn’t affect mRNA expression of either WT CHO-K1 or S. Confirming the observed rescue in the growth experiments described above, the expression of GS1 was upregulated in sGS5KO-K both in Gln-free medium and in the presence of MSX. Unexpected results were observed for sGS5KO-S clone as GS1 gene was also not expressed in this clone, even if only GS5 gene was deleted. The reason for this is unclear. This is because changes in gene expression are common in CHO cell subclones. Inhibition of GS1 expression could occur at the level of coding gene sequence, mutations in regulatory regions that may lead to altered expression, or epigenetic effects. It can not be stated with confidence whether the inhibition of GS1 expression is directly related to the KO of GS5 in CHO-S or occur during the selection of subclone.

### Evaluation of selection efficiency of GS-KO CHO cell lines during stable cell line generation

**Figure 5:**
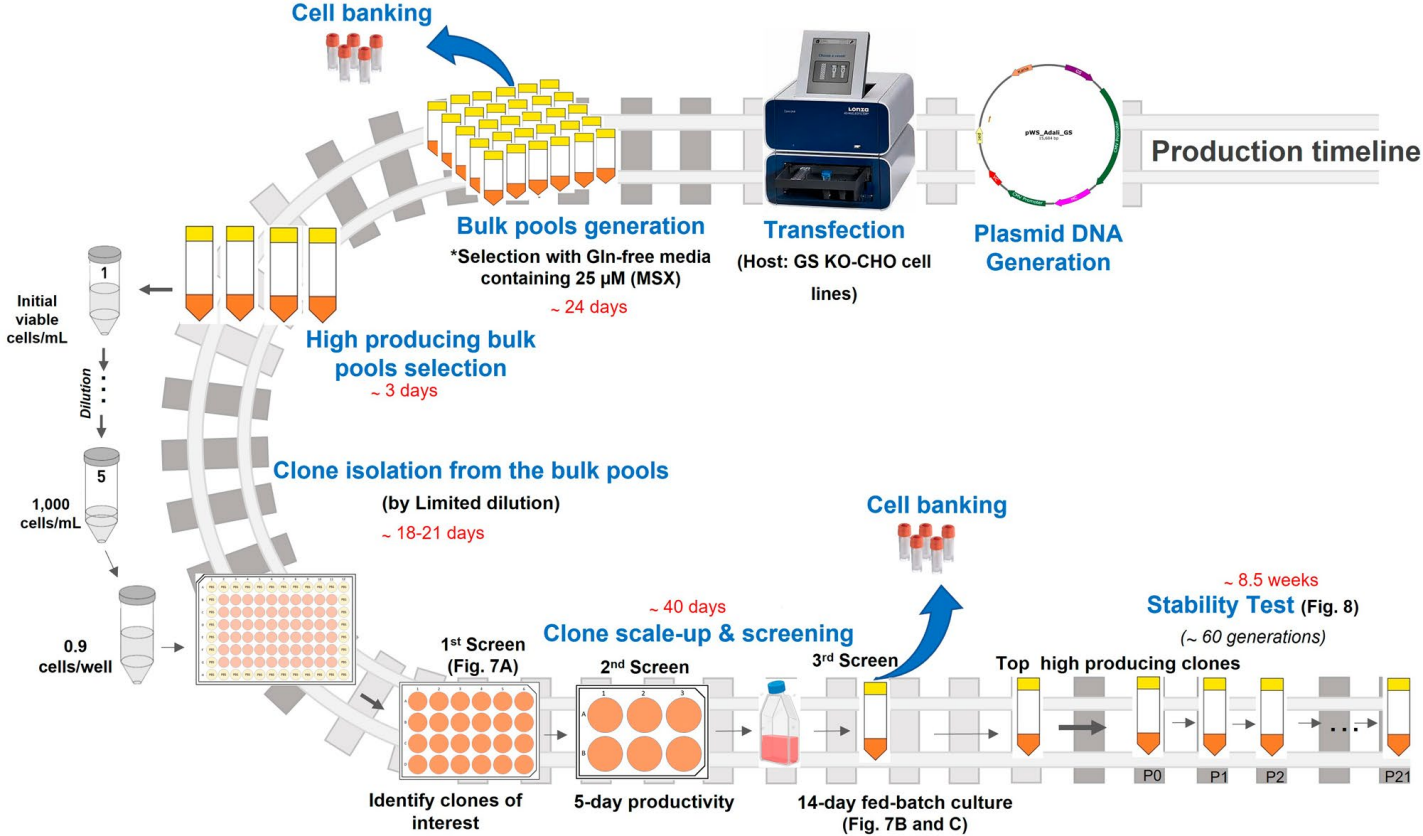
A workflow of the stable cell line generation process in this study. The figure illustrates major steps in clonal screening procedure and stability assessments of top clones. The mAb producing rCHO clones were created from different GS-KO CHO host cell lines generated in this study. A single round of selection with 25 µM MSX in Gln-free medium was conducted. Clones selected from different host cell lines were analyzed with respect to Qp and long-term production stability over more than 60 generations.

The efficiency of GS-KO CHO cells that have been created in this study for the generation of master cell banks for biologics manufacturing was evaluated according to the schematic diagram depicted in Fig. 5. The process starts with the transfection of a Freedom™ pCHO 1.0 expression vector containing two genes of optimized heavy (HC) and light (LC) chains of two model therapeutic Abs^29^ (Trastuzumab and Adalimumab). At 48 h after transfection, each bulk pool of transfected cells was centrifuged and resuspended in CD-CHO medium containing 0.2% Anti-Clumping agent without L-Gln (GS selection media). After the pools had recovered (viability > 90%), they were further selected with 25 µM MSX. The bulk pools were characterized for productivity and viability. The pool of each GS-KO CHO cell that showed the highest cell specific productivity (Qp) was used to generate single cells by limited dilution. The evaluation of each step of the stable cell line generation process is reported below.

**Figure 6:**
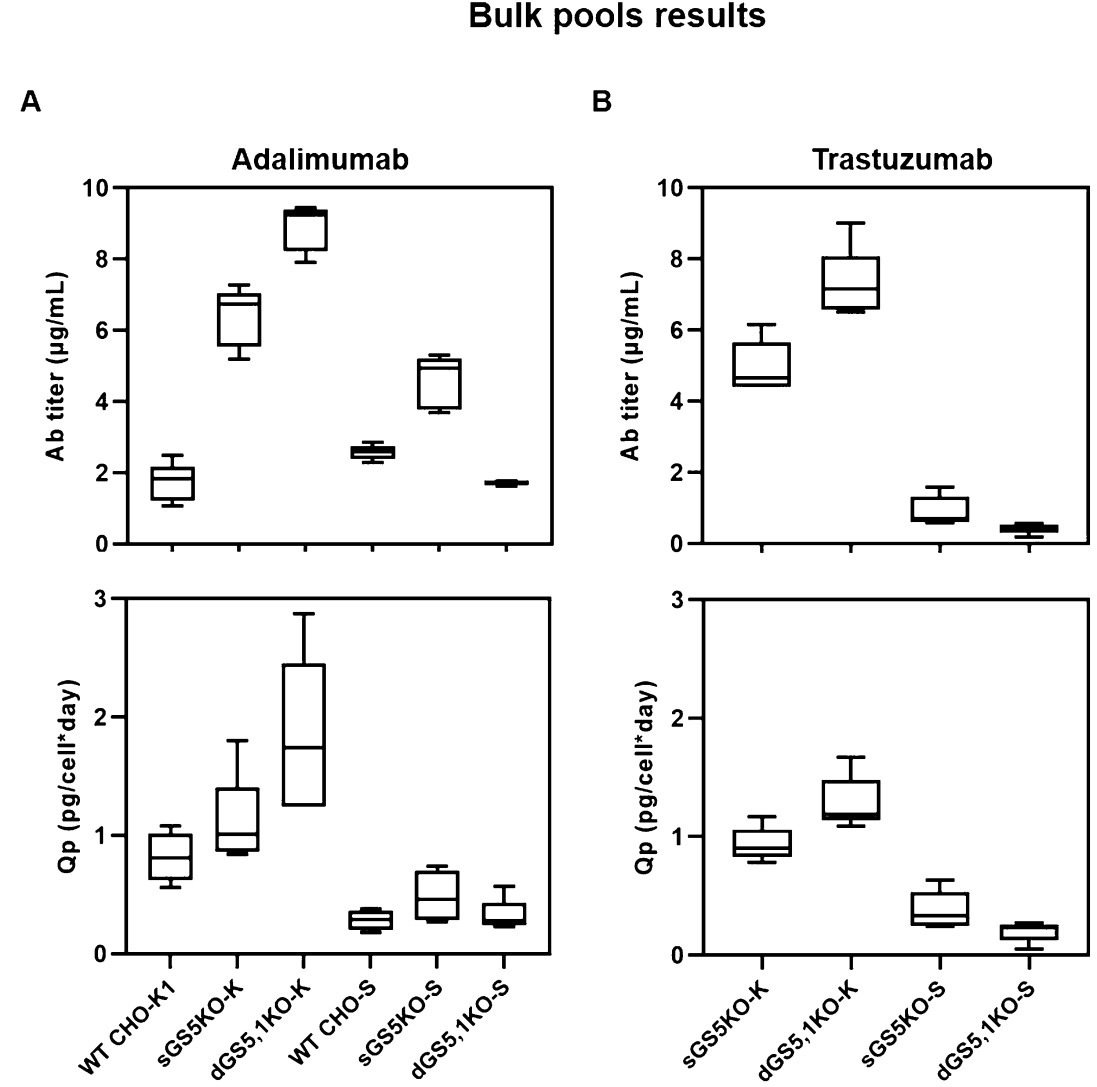
Ab titer and Qp of stable pools. The stable pools of each GS-KO CHO cell were monitored for Ab productivity during batch culture (after MSX selection). The Ab titer and the Qp of bulk pools for Adalimumab (**A**) and Trastuzumab (**B**) were determined on day 7. WT; wild type.

- **Stable pool generation.** The stringency of selection in the generated single and double GS-KO CHO-K1 and S cells was analyzed from a bulk culture of cells after Adalumimab transfection. In this experiment, the WT CHO-K1 and S cells were used as controls. No growth inhibition was observed when 25 µM MSX was added as previously reported^16^. After MSX selection, Ab productivity of a 7-day batch culture in the presence of 25 µM MSX conditions was monitored (Fig. 6A). The Ab titer and Qp of GS-KO CHO-K1-derived bulk pools was higher than those of the GS-KO CHO-S-derived bulk pools, with dGS5,1KO-K achieving the highest Ab titer and Qp. Among CHO-S cells, the sGS5KO-S-derived bulk pools revealed significant improvement in Ab production when compared to WT CHO-S, however, the dGS5,1KO-S-derived bulk pools fell short of expectation. These results indicated that sGS5KO-K, dGS5,1KO-K and sGS5KO-S cells had potential to be used for stable cell line generation with increased selection stringency to achieve higher Ab productivity.

To confirm the above findings, we repeated the stable cell line selection process using Trastuzumab^30^ (Fig. 6B). The result showed that bulk culture productivity profiles were similar to those of Adalimumab (Fig. 6B). After recovery from GS selection, intracellular staining of HC and LC of Trastuzumab during a 7-day batch culture in the absence of 25 µM MSX conditions were performed as shown in Supplementary Fig. S6 and S7. The dots in the upper right quadrant indicate good expression of both HC and LC in GS-KO CHO cells. These results suggest that high producing cell populations could be established in the absence of MSX, which is more desirable in pharmaceutical industry. In addition, this study demonstrated that a single round of low concentration (25 µM) of MSX selection is sufficient to generate stable bulk pools as has been previously reported^19^.

**Figure 7:**
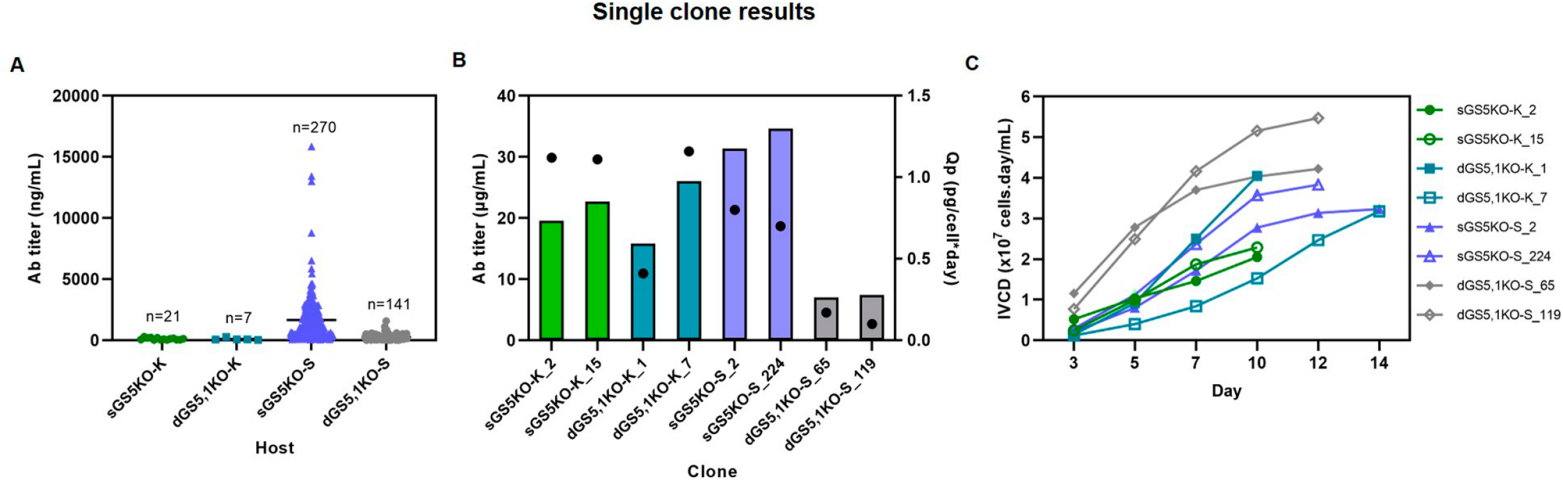
Screening of stable cell lines from different GS-KO CHO cell lines. (**A**) Ab concentration in culture supernatants in 24-well plates of individual single-cell clones derived from different GS-KO CHO cells. Ab productivity [Ab titer (bar) and Qp (black circle)] (**B**) and IVCD (**C**) of the top two highest-producing clones derived from each GS-KO CHO cell for a 14-day simple fed-batch culture are illustrated.

- **Single clone isolation.** The next step of stable cell line generation process is isolation of single cell subclones to create stable Ab producing clones. To evaluate the efficiency of generated GS-KO CHO cells after MSX selection, the highest producing pool of each GS-KO CHO cell line was subjected to standard limiting dilution to establish single cell-derived clones. These were successively expanded from 96-well plates to 24-well plates, 6-well plates, T-25 flasks, and then to 50 mL TPP tubes (Fig. 5). As shown in Supplementary Table S3, in the 24-well plate, the GS-KO CHO-K1 cells had lower cloning efficiency and percentage of Ab-producing clones than the GS-KO CHO-S cells. While for CHO-K1, double GS5,1-KO seemed to be better than single GS5-KO in terms of number of Ab-producing clones, the contrary was seen in CHO-S, as already observed on the pool level. For CHO-S, single GS5-KO cells were superior to double GS5,1-KO for both cloning efficiency and the percentage of clones that produced Ab. The Ab titer of individual single cell clones generated from different GS-KO CHO cells in the 24-well plates is shown in Fig. 7A, where 12 clones expressed more than 5000 ng/mL. After expansion to 6-well plates, the productivities were assessed (second screen) again. Top-10 high-producing clones from sGS5KO-K, sGS5KO-S, dGS5,1KO-S, and Top-5 of dGS5,1KO-K clones were chosen for a 14-day simple fed-batch culture with glucose-only feeding to identify the best clone for Adalimumab production. The Ab productivity and IVCD of the two highest-producing clones from each cell type are shown in Fig. 7B,C, respectively. Both single (sGS5KO-K_2 and 15) and double (dGS5,1KO-K_1 and 7) GS-KO CHO-K1 as well as single GS-KO CHO-S (sGS5KO-S_2 and 224) cells showed higher Ab titer and Qp than dGS5,1KO-S (65 and 119, Fig. 7B), while the later had the highest IVCD (Fig. 7C). Taken together, our results demonstrated that, with the exception of dGS5,1KO-S, all engineered cells generated from this study can be used as a host cell line for the production of therapeutic antibody. It is important to note that the glycosylation patterns of therapeutic antibody generated from different clones must be assessed as different patterns might be observed from different cell lines^31,32^. These issues are essential for antibody manufacturing especially for the production of biosimilars; however, they are highly subclone specific and thus beyond the scope of the current study.

### Stability assessment

**Figure 8:**
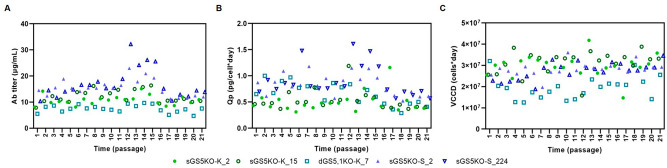
Time course of Ab productivity of 5 best-producer clones when long-term cultured in the absence of L-Gln and MSX. Ab concentration of the culture supernatant (**A**), Qp (**B**) and viable cumulative cell days (VCCD, (**C**)) as determined on days 7 of a batch culture.

Finally, clonal stability was assessed for representative stable clones for the different GS-KO CHO cells. Production stability of individual clones is the most important criteria for successful production of therapeutic Ab in industry due to the inherent genetic instability of CHO cells^33,34^,The stability of 5 best-producing clones, 2 from sGS5KO-K (2,15), 1 from dGS5,1KO-K (7), and 2 from sGS5KO-S (2,224), was assessed for Ab productivity during long-term cultures of 21 passages (over 60 cell generations) without L-Gln and MSX. Regardless of the host cell lines used, Ab productivity of all clones remained stable during long-term cultures. Their productivity after 60 generations was more than 80% of the respective original productivity (Fig. 8). The growth rate of all clones was also relatively stable (Fig. 8C). It has been reported that Ab productivity of some clones decreased when cells are cultured in medium without MSX^16^. This was not observed with our results.

In conclusion, an efficient and highly selective GS-KO CHO expression system, suitable for cell line generation could be established using a CRISPR-based full-gene-deletion strategy. The autologous expression level of GS genes in the respective host cell lines contributes to the stringency and in the case of CHO-K1 the upregulation of the second GS1 gene was demonstrated to prevent efficient selection of recombinant cells upon KO of only the GS5 gene. It may therefore be a good strategy to characterize precise expression of gene variants in the respective host before designing a knockout strategy also for potential future new selection markers.

## Methods

### Cloning of pgRNAs into CRISPR/Cpf1 vector

The gRNA sequences of 23 nucleotides (nts) targeting all 2 GS genes were extracted and designed with a Bowtie program developed by Langmead et al.^35^. The gRNAs were chosen only if the 27 nts frame the following criteria: (1) started with TTT[ACG]; (2) does not contain TTTT, TTTTT, GGGGG, AAAAA, CCCCC; (3) does not contain recognition sites for restriction enzymes *Bsm*bI and *Bbs*I (CGTCTC, GAAGAC); (4) individual GC content is between 0.3 and 0.7; (5) without off-targets across entire genome with 0 mismatch in seed. Each possible gRNA in the upstream region (on the left of the scaffold, irrespective of direction) was paired with gRNAs in the downstream region (on the right of the scaffold, irrespective of direction) along-with the scaffold sequences. Only the gRNAs in the frames that have GC content between 0.38 and 0.44 were selected (Supplementary Table S4). The selected pgRNAs were obtained as single-stranded (ss) oligonucleotides (Sigma-Aldrich, USA), annealed, and cloned into the pY010 (AsCpf1) vector^22^ according to Bauer et al.^27^. The sequences of recombinant plasmids were confirmed by Sanger sequencing (Eurofins, Austria) using the U6 promoter primer.

### Cell lines and cell culture

CHO-S (cGMP Banked, Cat. No. A13696-01), was purchased from Thermo Fisher Scientific (USA). CHO-K1 and S were maintained in CD-CHO medium (Thermo Fisher Scientific) supplemented with 8 mM L-Gln (Thermo Fisher Scientific, USA) and 0.2% Anti-Clumping Agent (Thermo Fisher Scientific, USA). Cells were grown in either TubeSpin Bioreactor 50 (TPP) tubes (TPP Techno Plastic Products, Switzerland) with a working volume of 15 mL or 125 mL shaking flasks (Corning, USA) with a working volume of 25–30 mL. The cell cultures in the tubes and shaking flasks were incubated at 37 °C, 7% CO_2_, and humidified air at a shaking speed of 250 and 140 rpm, respectively. The cells were passaged twice a week with a seeding density of 2 × 10^5^ viable cells per mL. The cell cultures were mixed with trypan blue and counted by ViCELL XR Cell Counter (Beckman Coulter, Germany) for VCD and % viability measurement.

### Cell transfection

For transfection, appropriate vectors were transfected into CHO-K1 or S cells by nucleofection as previously published^22^. In brief, 5 × 10^6^ viable cells were electroporated with 10–12 µg of circular plasmid DNA using a Neon Nucleofector device and the 100 µL Neon Transfection Kit (Thermo Fisher Scientific, USA) with 1700 V, 20 ms and 1 pulse. The transfected cells were transferred into a TPP tube containing 15 mL of pre-warmed medium and held in a static incubator at 37 °C, 7% CO_2_, and humidified air for 2 h. After that, the transfected cells were cultured under standard conditions. To check for transfection efficiency, the pY010 (AsCpf1) plasmid bearing a gene encoding green fluorescent protein (GFP) was transfected in parallel. After 24 h, transfection efficiencies were determined by CytoFLEX S flow cytometer at 488 nm excitation and 523 nm emission wavelengths (Beckman Coulter, Germany) to evaluate the percentage of GFP-expressing cells^29^.

### Assessment of pgRNA efficiency

To assess the efficiency of pgRNAs, the pY010 (AsCpf1) vector (Supplementary Fig. S8) containing different pgRNAs (3 µg each) were transfected into 2 × 10^6^ CHO-K1 and S cells. At day 5 post transfection, the genomic DNA (gDNA) was isolated with the DNeasy1 Blood & Tissue Kit (Qiagen, USA), according to the manufacturer’s protocol. The isolated gDNA was tested by deletion PCR with specific primers (Supplementary Table S5) using GoTaq^@^ G2 DNA polymerase (Promega, USA). The amplified DNA products were visualized of by agarose gel electrophoresis using Midori Green Advance (Biozym, Germany) for DNA staining and documentation with the Gel Doc™ XR system (Bio-Rad, USA). The pgRNAs that showed the deleted PCR products were selected for further GS gene deletion experiments.

### Fluorescent activated cell sorting (FACS)

GS-KO single cells were isolated by FACS (MoFlo Astrios Cell Sorter, Beckmann Coulter, USA). The transfected cells in 200 μL cultivation medium supplemented with L-Gln and 1 × penicillin/streptomycin (Sigma, USA) were sorted at single cell per well into 96-well plates and incubated at 37 °C in humidified air and 7% CO_2_. The single colonies were selected based on visual observation during colony growth and expanded into 48-well plates.

### Identification of GS-KO cell lines

For initial screening, samples for colony PCR were prepared following a previously published method^36^. The crude cell lysates were subjected to non-deletion PCR with specific primers (Supplementary Table S6). Lysates of positive clones (no non-deletion PCR band) were further evaluated by deletion PCR to confirm the deletion of the gene. The candidate clones were re-confirmed with non-deletion PCR and deletion PCR using pure gDNA as a template. All PCR reactions with either crude cell lysate or isolated gDNA were routinely performed using GoTaq^@^ G2 DNA polymerase (Promega, USA), following manufacturer’s instructions.

### DNA sequence analysis

Plasmids were prepared using a plasmid purification kit as suggested by the manufacturer (MiniPrep; Qiagen, USA). Automated DNA sequencing was performed by Eurofins, Austria, using U6 promoter primer. To analyze the deleted regions, the PCR products were amplified by Phusion High-Fidelity DNA Polymerase (Thermo Fisher Scientific, USA) for all genes with deletion primers as indicated in Supplementary Table S5 and purified with Monarch® PCR & DNA Cleanup Kit (NEB, USA), following manufacturer’s instructions. The purified PCR products were sequenced using deletion primers. The DNA sequences were analyzed with SnapGene version 4.2.6 (GSL Biotech, USA).

### Confirmation of GS genes deletion by RT-qPCR

To confirm GS gene deletion, total RNA was isolated from 1 × 10^6^ viable cells using Direct-zol RNA Kit (Zymo Research, USA) with DNase treatment, following manufacturer’s instructions. RNA (800 ng) was converted to cDNA using a High-Capacity cDNA Reverse Transcription Kit with RNase Inhibitor (Applied Biosystems, USA). For Quantitative PCRs (qPCRs), one microliter of the 4 × diluted cDNA was used as a template in 10 µL reaction using the SensiFAST SYBR Hi-ROX Kit (Bioline, UK). Samples were measured in quadruplicates on a Rotor-Gene qPCR cycler (Qiagen, Germany) as previously described^37^. The primer pairs specific for CHO, i.e., GAPDH (glyceraldeyde-3-phoshate dehydrogenase), Alpha-(1,6)-fucosyltransferase (FUT8) and three GS genes are listed in Supplementary Table S2. Relative quantification of gene expression level was normalized to GAPDH (for mRNA) or FUT8 (for gDNA) for calculation of fold change (FC) by $2^{-\Delta \Delta \text C_ \text T}$ method^38^.

### Assessment of impact on growth

The growth curve experiments were performed in TPP tubes with a working volume of 15 mL medium supplemented with or without 8 mM L-Gln. Each tube was inoculated in triplicates with a viable cell concentration of 2 × 10^5^ cells/mL and cultivated under standard conditions. Samples were taken daily to measure VCD, % viability and cell size.

### Transfection and selection of GS-KO cell lines expressing rAbs

To evaluate the selection efficiency of each engineered CHO cell, 5 sets of single and double GS-KO CHO-K1 and S were transfected with a linearized FreedomTM pCHO 1.0 vector (Thermo Scientific, USA) containing the optimized HC and LC genes of Adalimumab^29^ or Trastuzumab^30^, and the GS gene (*gs*) (pWS_Adali_GS expression vector for Adalimumab in Supplementary Fig. S9) using a SG Cell line 4D-Nucleofector® X Kit (Lonza, USA), according to the manufacturer’s instructions. For Trastuzumab, the engineered CHO cells were transfected using a Neon Nucleofector device. Two days post transfection, each bulk pool was centrifuged and resuspended in 10 mL of GS selection medium. The tubes were incubated with shaking under standard condition. After the pools were recovered from GS selection (viability > 90%), the bulk pools were further selected with Gln-free medium supplemented with 25 µM MSX (Sigma-Aldrich, USA).

### Characterization of stable pools

The bulk pools were seed at 3 × 10^5^ cells/mL into TPP tubes containing 15 mL GS selection medium supplemented with 25 µM MSX. Samples were taken on day 7 post inoculation for VCD and Ab titre analysis. For Trastuzumab, the bulk pools were also characterized in GS selection medium without MSX.

### Determination of Ab titre

Ab titre of Trastuzumab was determined from cell supernatant by the Octet RED96e (ForteBio, USA) with protein A probes using human IgG1 Trastuzumab as a standard. For Adalimumab, Ab titer was assayed by ELISA according to previously published protocol with some modifications^39^. Nunc-Immuno 96-well plates were coated with 100 µL of 250 ng/mL Protein A (GenScript, USA) in PBS per well. After incubation at 4 °C overnight, the plates were washed 3 times with PBST (0.05% Tween 20 in PBS), followed by blocking with 2% MPBS (2% skimmed milk in PBST) at room temperature for 1 h. One hundred microliters of the optimal dilution of supernatant and various concentrations of antibody standard (Humira®), ranging from 250 to 0 ng/mL in PBS, were added to each well and incubated at room temperature for 1 h. The wells were washed three times with PBST. Abs were detected with 1:5,000 dilution in 2% MPBS of Peroxidase AffiniPure F(ab’)2 fragment Goat-anti human IgG (H + L) HRP (Jackson Immuno Research Laboratories, USA). Color reactions were developed using ABTS (2, 2-azino-di-3-ethyl-benzothiazoline-6-sulfonate) peroxidase substrate (VWR, Singapore) containing 0.05% H_2_O_2_. After the reaction was stopped with 1% SDS, the absorbance (OD) at 405 nm was measured with an ELISA plate reader (Sunrise, TECAN, Austria).

### Determination of Qp

To assess the Qp (pg/cell/day), the VCD and Ab titer were evaluated and Q_p_ was calculated as previously described^40^ with some modifications. Integral viable cell density (IVCD) was calculated according to Eq. (1) where *x*_*t0*_ and *x*_*t*_ represent the total viable cell concentration (cells/mL) at the beginning and end points, IVCD_*t0*_ and IVCD_*t*_ are IVCD at the beginning and end points, and Δ*t* is time interval in days.

1 $${\mathrm{IVCD}}_{t}=\frac{({x}_{t}+{x}_{{t}_{0}})}{2}\times \Delta t+{\mathrm{IVCD}}_{{t}_{0}}$$

Qp was calculated according to Eq. (2) where ∆*P* (pg) and ∆IVCD are the change in Ab amount and IVCD over the cumulative time interval of the evaluation, *t*-*t*_*0*_.

2 $$\mathrm{Qp} =\frac{\Delta P}{\Delta \mathrm{IVCD}}$$

### Single clone isolation

- **Primary and secondary screening.** To obtain single cell clones, the bulk pools that showed the highest productivity from each engineered CHO cell were subjected to limiting dilution using GS selection medium. First, the clones were allowed to grow in their 96-well plates, then an appropriate number of clones from each group were transferred into 24-well plates. After identifying clones of interest by ELISA, the clones were scaled up into the next larger plate and vessel (i.e., 24-well plates to 6-well plates, to T-25 flasks, and then to 50-mL TPP tubes). Next, secondary screening was performed to identify top-10 producing clones from each group. In this screening, the cells at 3 × 10^5^ viable cells/mL were seeded into 6-well plates containing 3 mL of GS selection medium supplemented with 3 g/L glucose and incubated without shaking for 5 days before sampling for productivity. The top-producing clones were counted and passaged at least two times into TPP tubes containing 15 mL of GS selection medium before starting the tertiary screen to ensure that the doubling times have stabilized.
- **Tertiary Screening.** To identify the best producing clones from each group for the next stability assessment step, tertiary screening was performed based on a 14-day simple fed-batch with glucose-only feeding. The cells at 3 × 10^5^ viable cells/mL were seeded into TPP tubes containing 15 mL of GS selection medium and incubated with shaking at 200 rpm until culture viability dropped below 50% or day 14 of cultivation was reached. The cell culture supernatants were sampled at regular intervals (e.g., on days 0, 3, 5, 7, 10, 12, and 14) to determine VCD, % viability, and Ab titer. The cell cultures were fed with 4 g/L of glucose (on day 3 and 5) and 6 g/L of glucose (on day 7) after sampling.

### Stability assessments of top clones

Each selected CHO single clone expressing rAb was seeded at 2 × 10^5^ viable cells/mL in a TPP tube containing a working volume of 15 mL Dynamis™ Medium, 0.2% Anti-Clumping agent (without L-Gln), and incubated at 37 °C, 7% CO_2_, and 80% humidity with shaking at 200 rpm. The clones were passaged every 3 days. The VCD and % viability of cells in the cultures was determined on days 3 and 7. After passaging into the TPP tube, the cells remaining in the old TPP tube were fed with 5 g/L glucose, and continued incubation for 7 days before the culture supernatant was sampled to determine the concentration of the secreted Ab by ELISA and clonal productivity. The stability analysis was carried out for 21 passages (over 60 cell generations).

## Supplementary Information

Supplementary Information.

## Data Availability

All data generated or analyzed during this study are included in this published article and its Supplementary Information files. FACS data generated during this study are available in the FlowRepository 1) FR-FCM-Z5VX (http://flowrepository.org/id/RvFrKdPH6p8gqun1JpMP6RDgUilLpJrAvOVJcqpjgumj10wFZgXOBGaTvgxjSeov), and 2) FR-FCM-Z5VN (http://flowrepository.org/id/RvFriuvtFucnWuV6BCqdwBnBA3rgFw0qXmer4sQB1PBufU4ZY4SJYIivpi85jqKV). Flow cytometry analysis data generated during this study are available in the 1) FR-FCM-Z5VK (http://flowrepository.org/id/RvFrnegPLMMFQSwuAtjqyrrPbh0WALtXSUN8xQZTBEm8Ok7Wd0CMBfVuaVBr3kve), and 2) FR-FCM-Z5VL (http://flowrepository.org/id/RvFruVCrGSPfLo93iRRR4t1T8iECgVqvsXtRY1G0XFydeAIQTA4EFGHxHNXeAMVb). GenBank Accessions of sequences in this study are listed as follows; OP895119.1: glutamine synthetase (GLUL) mRNA, OP892520.1: Adalimumab immunoglobulin heavy chain, OP892521.1: Adalimumab immunoglobulin kappa light chain, OP892522.1: Trastuzumab immunoglobulin heavy chain, OP892523.1: Trastuzumab immunoglobulin kappa light chain. Graph and Statistic analysis was performed using GraphPad Prism 8.0.1 software (https://www.graphpad.com/updates/prism-801-release-notes?fbclid=IwAR0SMvrkrXR6EAnVoV3klghG7Wa614o519u0CEcfYmzDDEeL5OVF6KOUdpo).
