# Supplementary material for: Glutamine synthetase (GS) knockout (KO) using CRISPR/Cpf1 diversely enhances selection efficiency of CHO cells expressing therapeutic antibodies
Source: Sci Rep. 2023 Jun 28;13:10473. doi: 10.1038/s41598-023-37288-6 (PMC10307828; doi:10.1038/s41598-023-37288-6)
Supplement: Supplementary file 1 — Supplementary Information. [file 41598_2023_37288_MOESM1_ESM.pdf]

Supplementary Information for

Glutamine synthetase (GS) knockout (KO) using CRISPR/Cpf1 diversely enhances selection efficiency of CHO cells expressing therapeutic antibodies

Witsanu Srila[1], Martina Baumann[2], Markus Riedl[2,3], Kuntalee Rangnoi[1], Nicole Borth[2,3*] and Montarop Yamabhai[1*]

[1]Molecular Biotechnology Laboratory, School of Biotechnology, Institute of Agricultural Technology, Suranaree University of Technology, Nakhon Ratchasima, Thailand.

[2]Austrian Centre of Industrial Biotechnology (ACIB), Vienna, Austria.

[3]Department of Biotechnology, University of Natural Resources and Life Sciences (BOKU), Vienna, Austria.

*Corresponding author

E-mail: montarop@g.sut.ac.th and nicole.borth@boku.ac.at

This file contains 1 supplementary information, 9 supplementary figures and 6 supplementary tables.

Supplementary information 1

Bioinformatic analysis of GS genes in CHO-K1 cells

A search and analysis of the CHO genome databases, assembly CriGri_1.0 (GCF_000223135.1), revealed two GS genes: glutamate-ammonia ligase (Glul; GS5), transcript variant X1 (location: NW_003613921.1 (1427530-1436859)) and LOC100689337 (Glul pseudogene; GS1; location: NW_003614063.1 (177163-178284)). When double checked from assembly CriGri-PICRH-1.0 (GCF_003668045.3), two GS genes were found: Glul transcript variant X2 and 3 (GS5; location: chromosome 5: NC_048598.1| 37796573-37806392, complement), and LOC100689337 (GS1; Glul pseudogene; NW_023276807.1|167206419-167207540), which corresponds to a search in CGR-Reference genome: https://cgr-referencegenome.boku.ac.at/. While the sequence identified in previous, less perfect assembly in Chromosome 6 appears to be an assembly artefact.

Supplementary Figure S1. Evaluation of pgRNA efficiency for GS gene deletions. Each vector carrying pgRNA was transfected into CHO-K1 or sGS5KO-S or sGS5KO-K. After transfection, the transfected cells were tested for gene deletion by PCR. The pool cells showing the deletion amplicon indicated that the designed sgRNA pairs can be used to create GS-KO CHO cells. The illustrated gel is the representative example of one biological replicate.

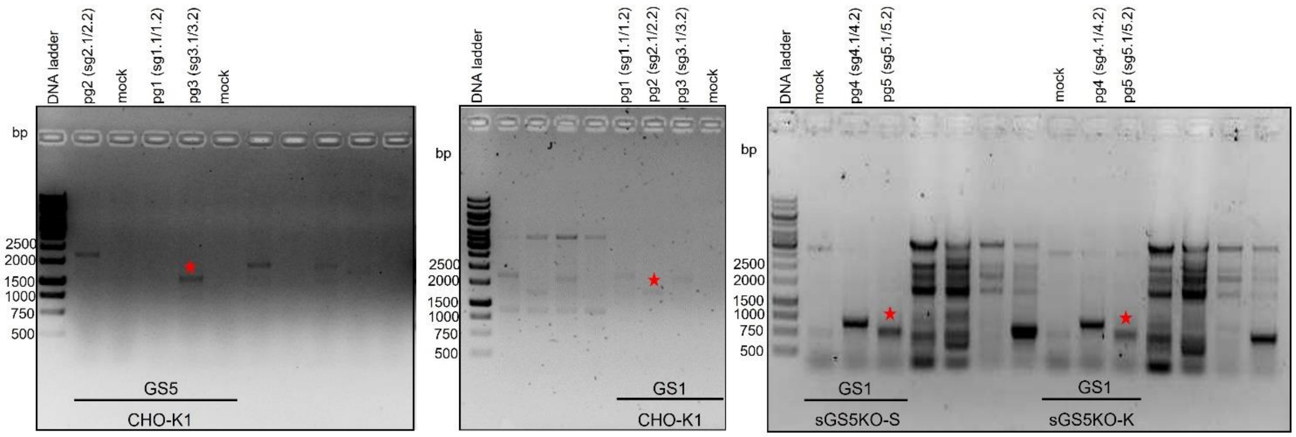

Supplementary Figure S2. Representative gel identifying biallelic clones. The non-deletion amplicon ~902 bp (A) and the deletion amplicon ~1096 bp (B) of individual clones are shown. The two biallelic deletion clones are clones S8 (CHO-S P27E; sGS5KO-S) and K3 (CHO-K1 P27D; sGS5KO-K) which marked with asterisk. As seen in the sequencing data, the deletion band for clone K3 has a smaller size due to larger deletions at the deletion junction. The GeneRuler 1 kb DNA Ladder (Thermo Scientific, USA) was used as a DNA marker. S; CHO-S clone and K; CHO-K1 clone.

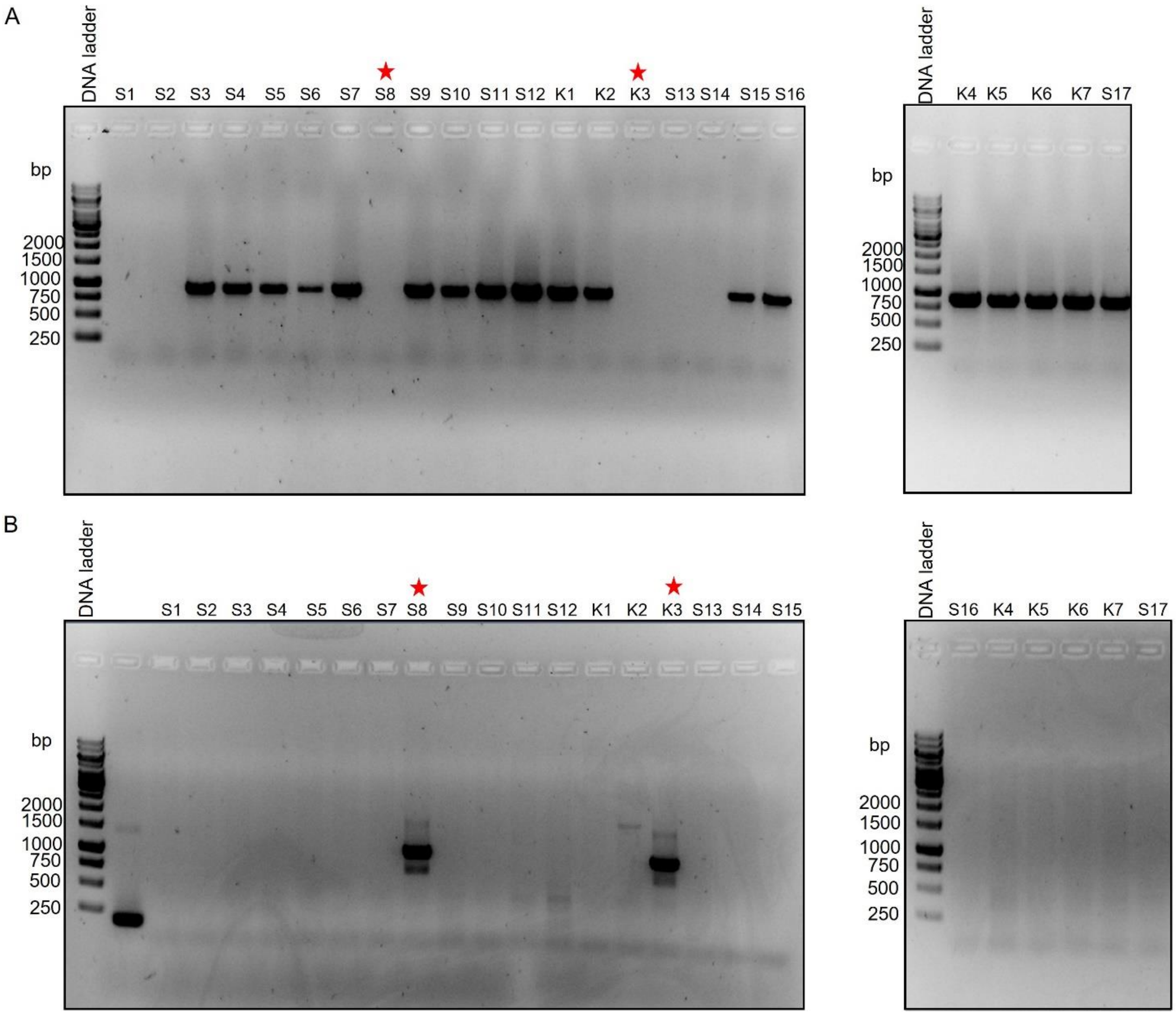

Supplementary Figure S3. Sanger DNA sequencing of GS-KO CHO cell lines. (A) DNA sequencing indicating that GS5 was deleted from CHO-K1 and CHO-S cells, resulting in sGS5KO-K (CHO-K1 P27D) and sGS5KO-S (CHO-S P27E). (B) DNA sequence analysis confirms that GS1 was successfully deleted from sGS5KO (single GS5 knockout) CHO cells, resulting in dGS5,1KO-K (CHO-K1 P1F3) and dGS5,1KO-S (CHO-S P2E5) clones.

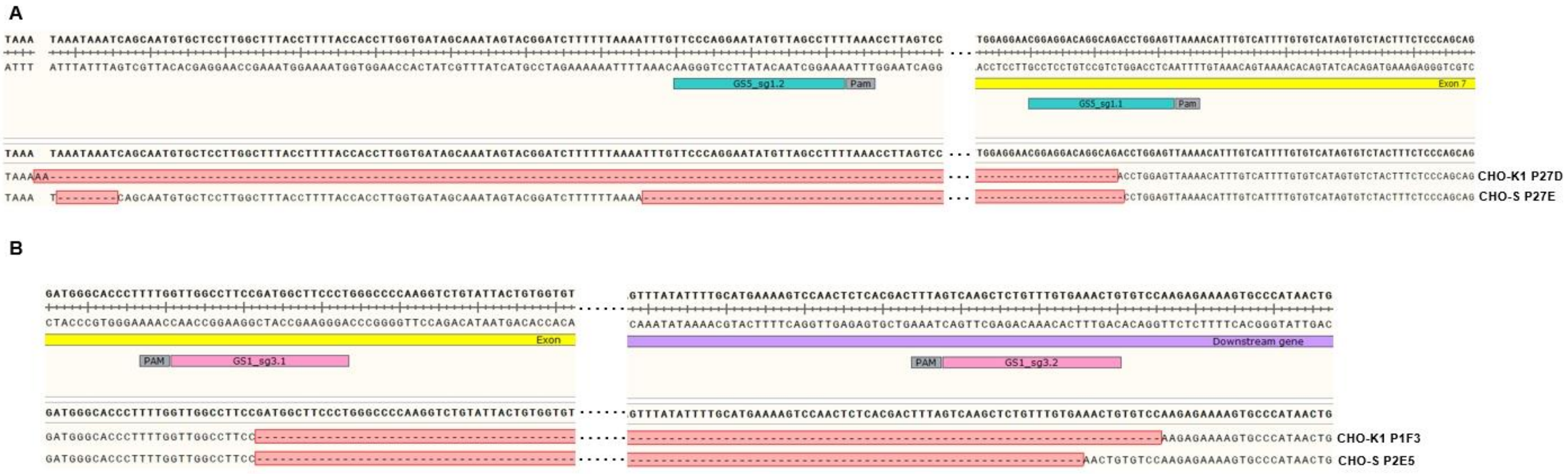

Supplementary Figure S4. Gel electrophoresis profile of transfected sGS5KO-K and sGS5KO-S cells with different pRNAs. Individual vector carrying pgRNA was transfected into sGS5KO-K and sGS5KO-S cells. After transfection, the transfected cells were tested for gene deletion by deletion PCR. The pool cells showing the deletion amplicon indicated that the designed sgRNA pairs can be deleted GS genes. The illustrated gel is the representative example of one biological replicate. The GeneRuler 1 kb DNA Ladder (Thermo Scientific, USA) was used as a DNA marker.

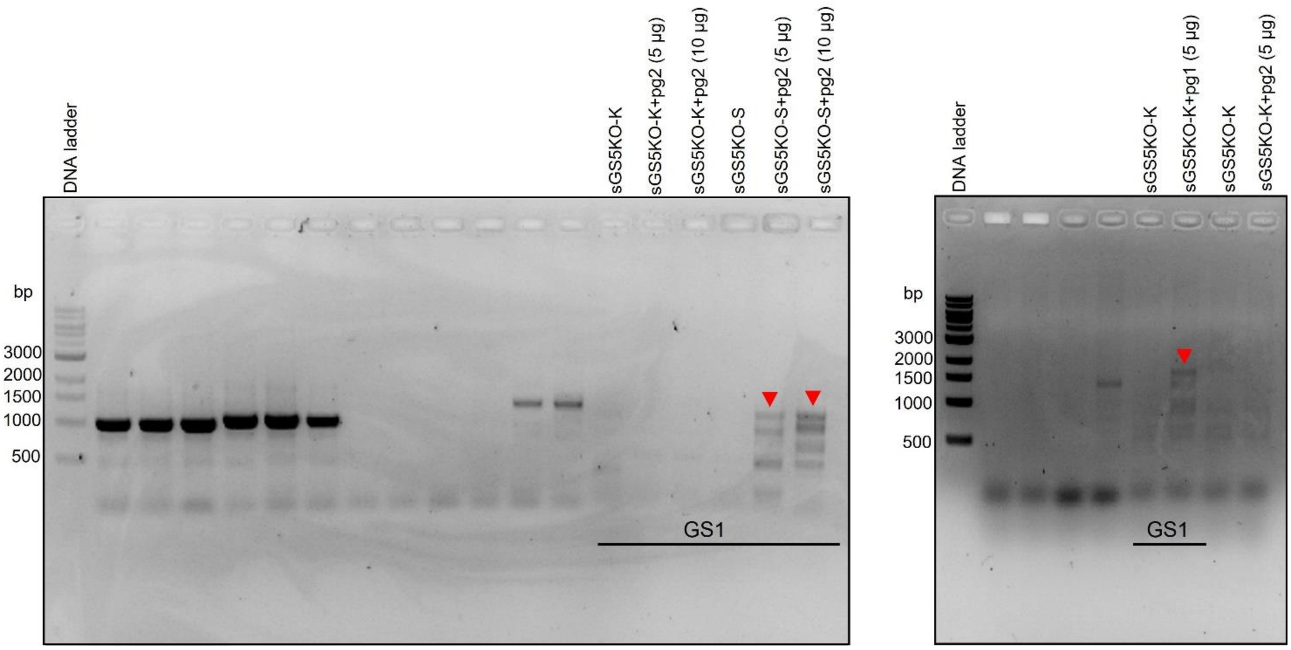

Supplementary Figure S5. Representative gel identifying biallelic clones. The non-deletion amplicon ~550 bp (A) and the deletion amplicon ~591 bp (B) of individual clones are shown. The six biallelic deletion clones are clones S2; P1A3, S5; P1F5, S6; P2E5, S9; P3F6, S10; P2A4 and K6; P1F3 which marked with asterisk. As seen in these figures, the deletion band for clones S9 and 10 have a difference in size due to larger or smaller deletions at the deletion junction. The 100 bp DNA Ladder (NEB, USA) was used as a DNA marker. S; CHO-S clone and K; CHO-K1 clone.

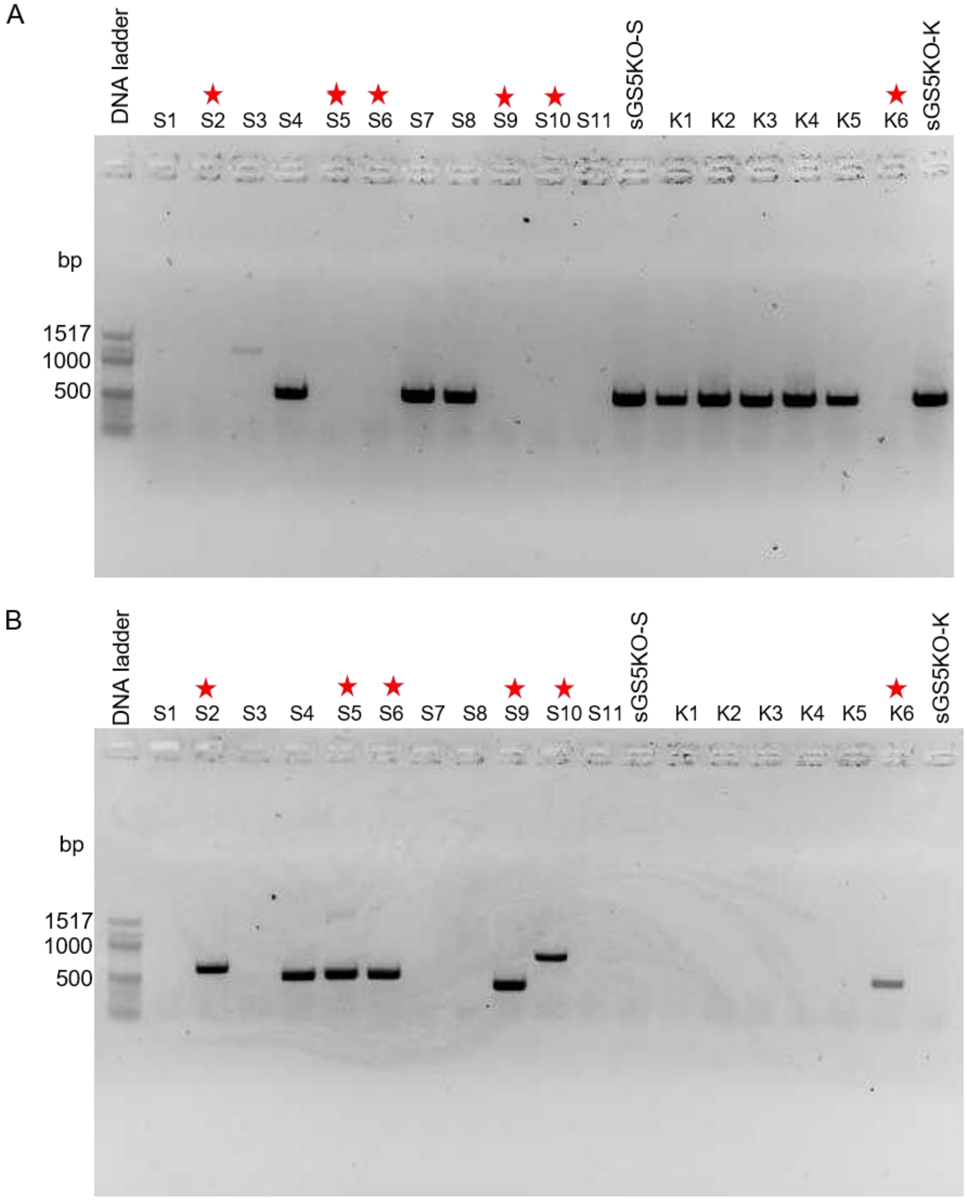

Intracellular staining

Ab-expressing CHO cells were stained following previously published method[1]. The cells expressing the Ab gene were stained with Anti-Human IgG (γ-chain specific) Ab labelled with R-Phycoerythrin (Cat. No. P9170, Sigma-Aldrich, USA) and anti-Human kappa light chain Ab labelled with FITC (Cat. No. F3761, Sigma-Aldrich, USA). The fluorescent signal intensity was measured using a CytoFLEX S flow cytometer (Beckman Coulter, Germany) and the data were analysed using the CytExpert 2.4.0.28 software program (Beckman Coulter). The experiments were performed in five biological replicates.

Supplementary Figure S6. Intracellular staining of Trastuzumab from the bulk cultures of GS-KO CHO-S cells. After the cell viability > 90% from GS selection, the bulk pools were further characterized in Ab production during batch culture. The cell pools were cultured GS selection medium without MSX and were taken for intracellular staining on day 7 post inoculation. sGS5KO-S (CHO-S P27E); dGS5,1KO-S (CHO-S P2E5).

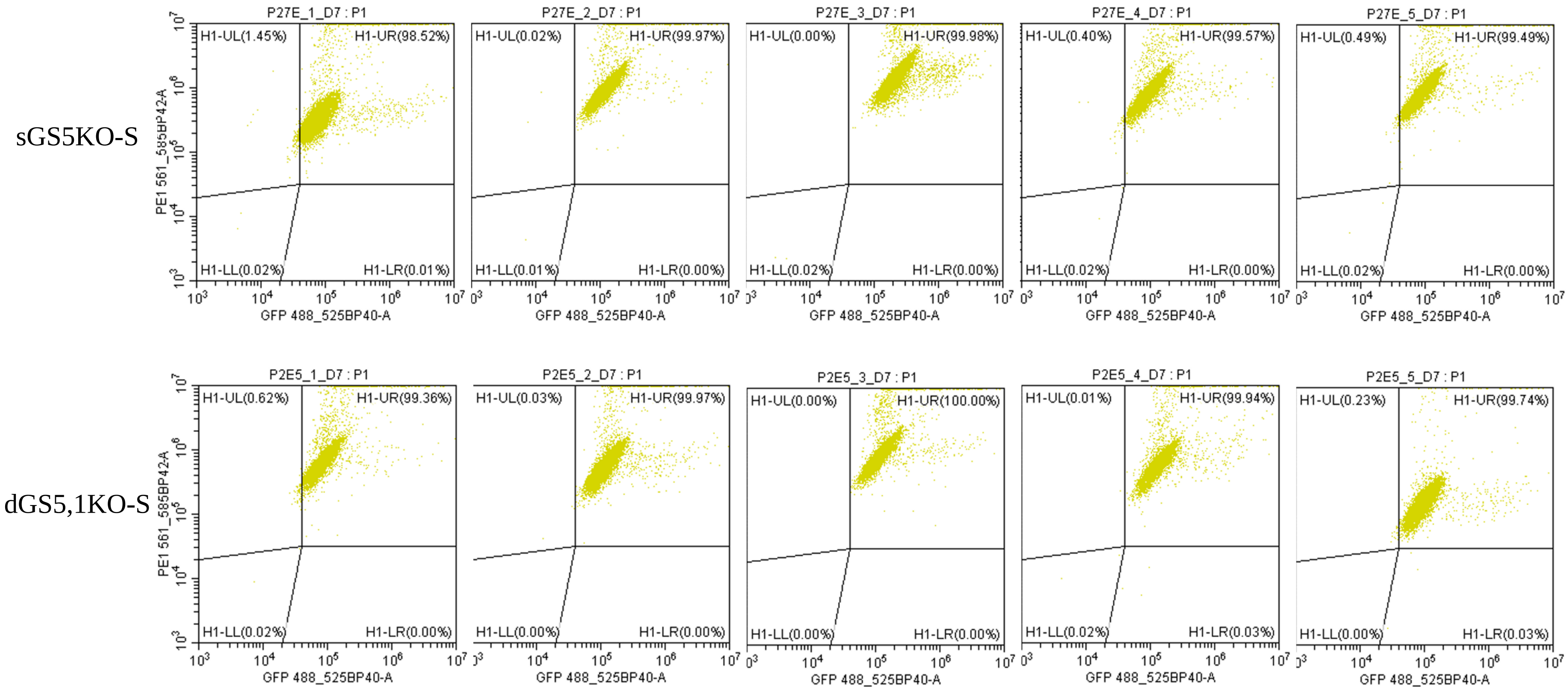

Supplementary Figure S7. Intracellular staining of Trastuzumab from the bulk cultures of GS-KO CHO-K1 cells. After the cell viability > 90% from GS selection, the bulk pools were further characterized in Ab production during batch culture. The cell pools were cultured GS selection medium without MSX and were taken for intracellular staining on day 7 post inoculation. sGS5KO-K (CHO-K1 P27D); dGS5,1KO-S (CHO-K1 P1F3).

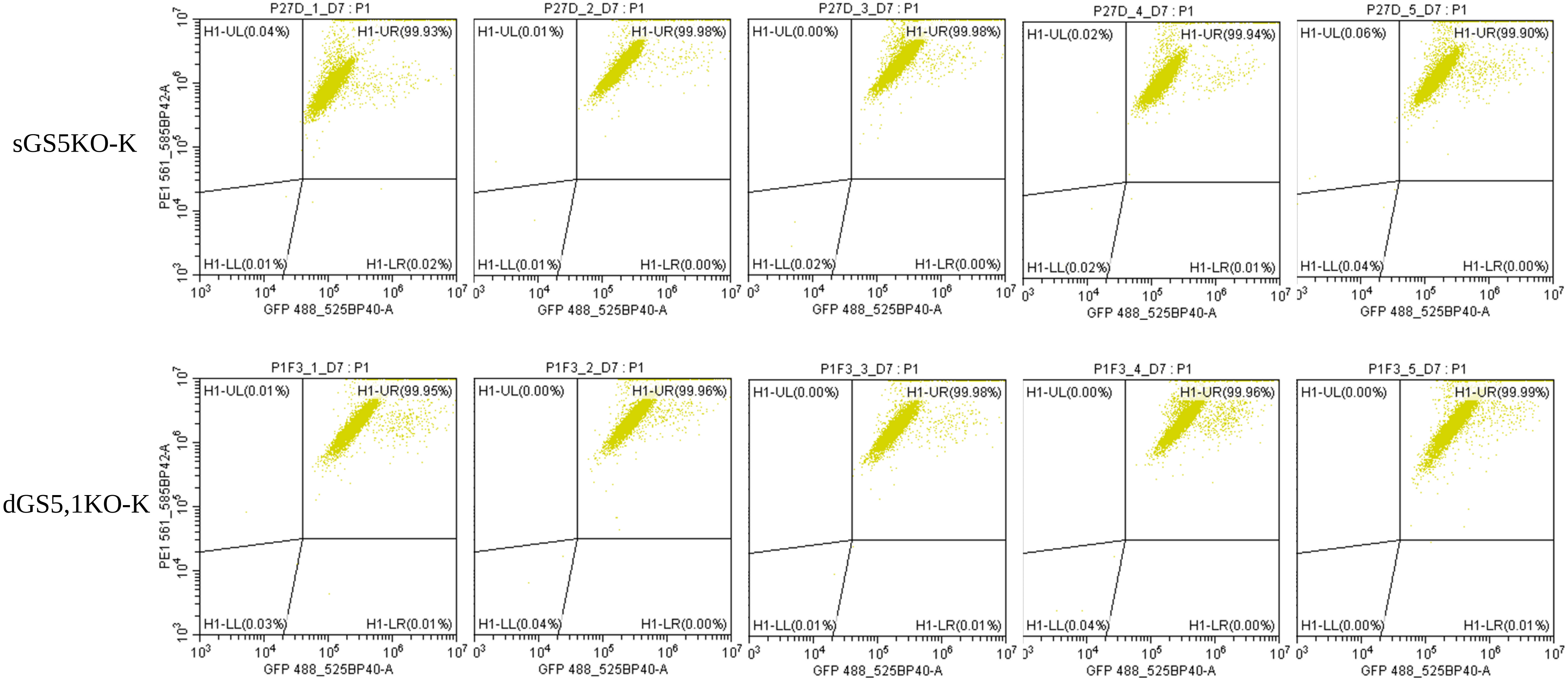

Supplementary Figure S8. Illustration of all-in-one pY010(AsCpf1)_eGFP_pgRNA vector[2]. This plasmid which contains one cassette for AsCpf1 as well as eGFP and a U6 promoter driven cassette for pgRNA expression, was used to introduce genetic deletions.

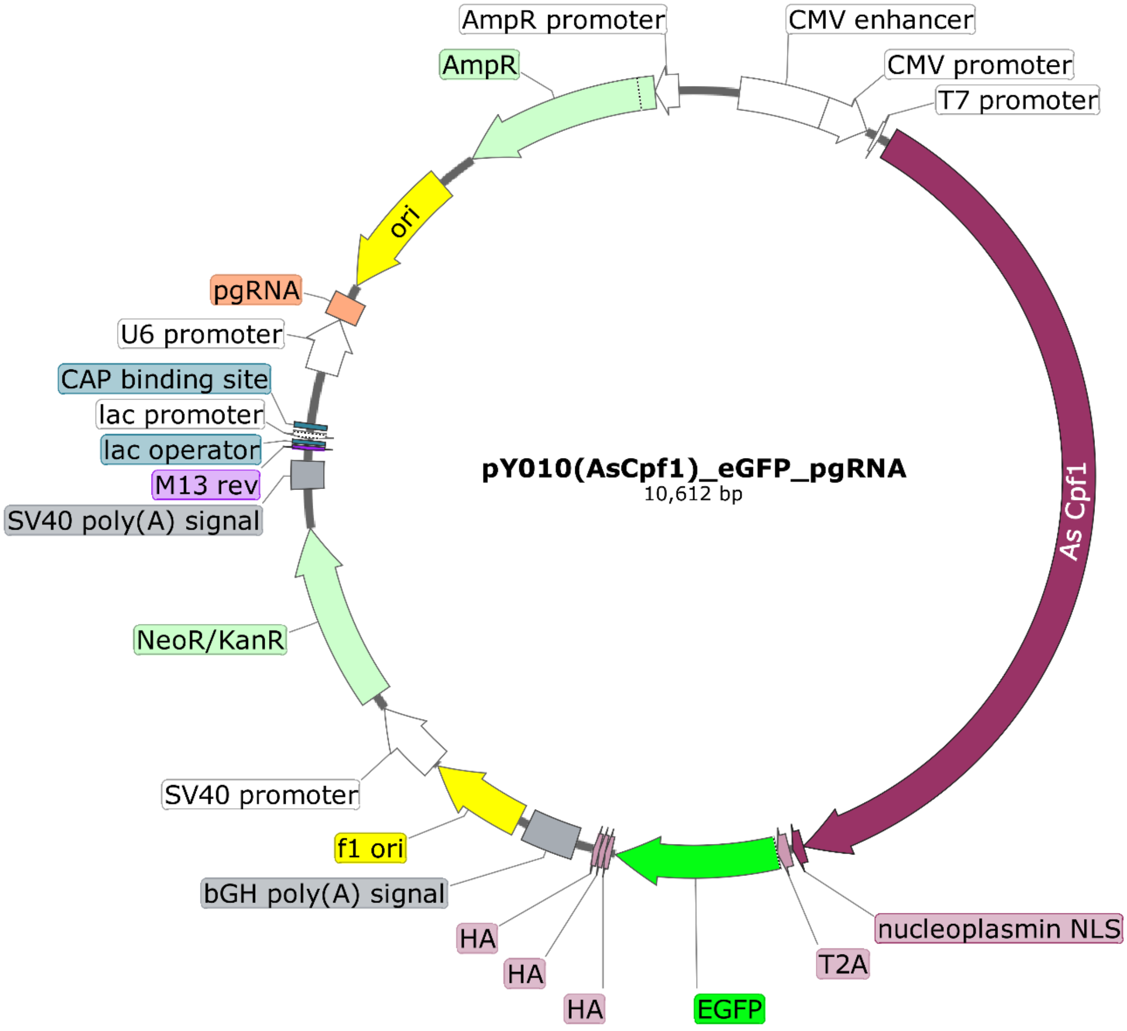

Supplementary Figure S9. Map of pWS_Adali_GS expression vector. This vector contains optimized heavy chain (HC) and light chain (LC) of Adalimumab with signal peptides, of which both genes were under the control of the cytomegalovirus (CMV) hybrid promoter and fused with signal peptide. The vector also carries the GS gene, under the control of the SV40 promoter, for selection and maintenance of the GS-KO CHO cells.

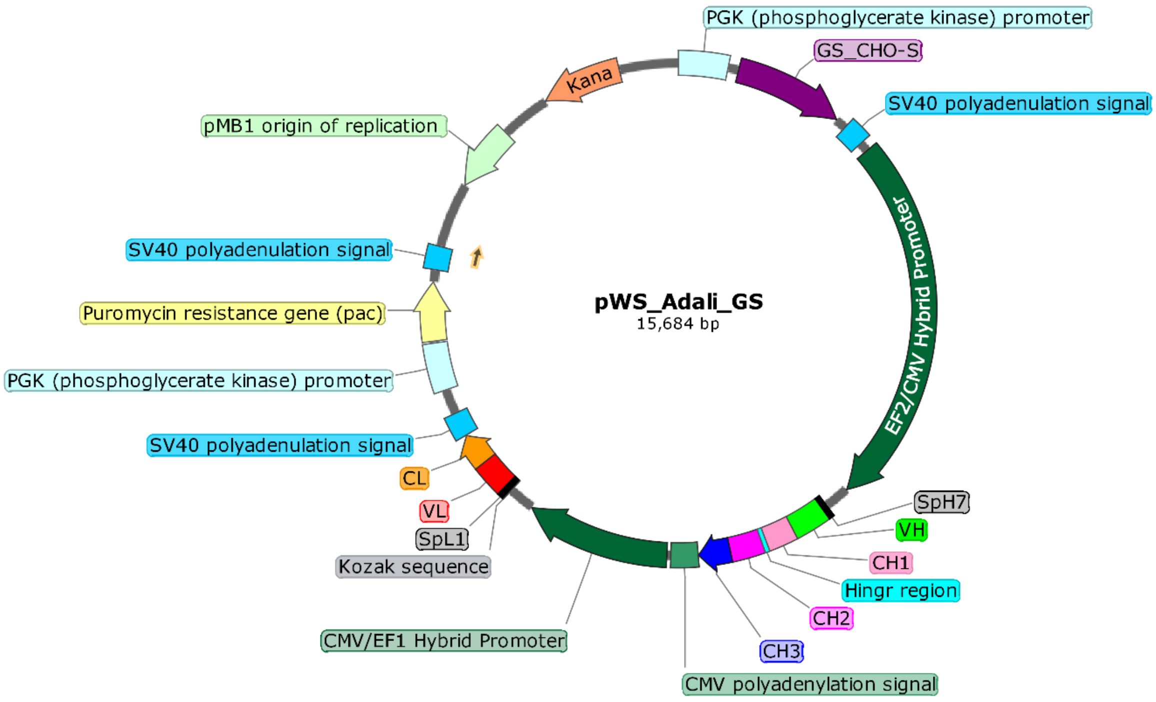

Supplementary Table S1. List of publications supporting our in-house RNAseq information on the expression of GS genes.

Accession_Number	DOI	Cell_Line	Cell information
ERP122753	http://doi.org/10.1016/j.csbj.2020.11.008	CHO-K1	Horizon Discovery CHO-K1 GS-/-
SRP066848	http://doi.org/10.1128/mBio.02128-16	CHO-K1	ATCC CHO-K1
SRP069883	http://doi.org/10.1016/j.cels.2016.10.020	CHO-S	CHO-S Life Technologies
SRP111368	http://doi.org/10.1002/biot.201700231	CHO-K1	CHOZN GS-/-
SRP159459	http://doi.org/10.1038/s41598-019-45126-x	CHO-K1	
SRP234382	http://doi.org/10.1002/bit.27365	CHO-K1	mAb-producing CHO-K1
SRP246348	http://doi.org/10.1038/s41467-020-15866-w	CHO-S	CHO-S Life Technologies
SRP324587	http://doi.org/10.1016/j.nbt.2022.01.010	CHO-K1	CHO-K1 AstraZeneca; MSX selection
ERP120293	http://doi.org/10.1016/j.csbj.2020.05.020	CHO-K1	ECACC CHO-K1 & CHO-K1 Hy (Cytiva)
ERP140863	http://doi.org/10.1002/biot.201700231	CHO-K1	ECACC CHO-K1

Supplementary Table S2. Primers used for qPCR including further specifications.

Name	Sequence (5'-3')	Annealing temperature (°C)	Target
qGS5_cpf1_Fw	TTAAGGCACTCGTGTAAACGG	60	GS5
qGS5_cpf1_RV	GAAAGCCATTGGAAGGCCAAC		
qGS6_cpf1_Fw	CCAAGCCCATTCCTGGGAAATA	60	GS6
qGS6_cpf1_Rv	GCTTCAGACCATTTTCCTCCCA		
qGS1_cpf1_Fw	ACTGGGTTCCACAAAACGTC	60	GS1
qGS1_cpf1_Rv	GATGGCTTCTGTCACTGCAA		
qFUT8_E7_Fw	GCTGTGGCTATGGATGTCAA	60	FUT8
qFUT8_E7_Rv	GTGCATGTCTCACTTACAGGTC		
qGAPDH_Fw	GAAAGCTGTGGCGTGATGG	60	GAPDH
qGAPDH_Rv	ACACGTTGGGGGTAGGAACA		

Supplementary Table S3. The percentage of cloning efficiency and Ab-producing clone of each GS-knockout CHO cell line obtained from first screening.

Cell	Total clone	Positive clone	% Cloning efficiency	% Ab-producing clone
sGS5KO-K	21	14	2.19	66.67
dGS5,1KO-K	7	5	0.73	71.43
sGS5KO-S	270	269	28.13	99.63
dGS5,1KO-S	141	121	14.69	85.82

Supplementary Table S4. The paired gRNA sequences including additional information for AsCpf1.

Name	Sequence (5'-3')	PAM gRNA1 (5'-3')	PAM gRNA2 (5'-3')	Target
pg1_Fw	caccGTAATTTCTACTCTTGTAGATTAAGCGCTGCTCTGGCAGTTACAAATTTCTACTCTTGTAGATAAGGTGAGACAAGGGGACTAAGGTTTTTGCG	TTTA	TTTA	GS5
pg2_Fw	caccGTAATTTCTACTCTTGTAGATCAGTAGGGCTACCCATCTGTCAGAATTTCTACTCTTGTAGATACCTTAACCCGTGATTTGTCTCCTTTTTGCG	TTTA	TTTA	
pg3_Fw	caccGTAATTTCTACTCTTGTAGATACTCCAGGTCTGCCTGTCCTCCGAATTTCTACTCTTGTAGATAAAGGCTAACATATTCCTGGGAATTTTTGCG	TTTA	TTTA	
pg1_Rv	aaacCGCAAAAACCTTAGTCCCCTTGTCTCACCTTATCTACAAGAGTAGAAATTTGTAACTGCCAGAGCAGCGCTTAATCTACAAGAGTAGAAATTAC	TTTA	TTTA	
pg2_Rv	aaacCGCAAAAAGGAGACAAATCACGGGTTAAGGTATCTACAAGAGTAGAAATTCTGACAGATGGGTAGCCCTACTGATCTACAAGAGTAGAAATTAC	TTTA	TTTA	
pg3_Rv	aaacCGCAAAAATTCCCAGGAATATGTTAGCCTTTATCTACAAGAGTAGAAATTCGGAGGACAGGCAGACCTGGAGTATCTACAAGAGTAGAAATTAC	TTTA	TTTA	
pg1_Fw	caccGTAATTTCTACTCTTGTAGATTCTACATTTTCTCTGGGCTAAGCAATTTCTACTCTTGTAGATGTACCAAGACCATGCGGGAGGAGTTTTTGCG	TTTA	TTTA	GS1
pg2_Fw	caccGTAATTTCTACTCTTGTAGATGAATTCCGTTCATCATACCCATAAATTTCTACTCTTGTAGATAAGCCCGCTGCCCCTCTGCCAATTTTTTGCG	TTTA	TTTG	
pg3_Fw	caccGTAATTTCTACTCTTGTAGATTTCTACTGTGAGTTGGCCCTTATAATTTCTACTCTTGTAGATGTTGGCCTTCCGATGGCTTCCCTTTTTTGCG	TTTA	TTTG	
pg4_Fw	caccGTAATTTCTACTCTTGTAGATAGACACACGTGTAAACGGATAATAATTTCTACTCTTGTAGATGGATCCTGCCTGTATCCTGTGAGTTTTTGCG	TTTA	TTTA	
pg5_Fw	caccGTAATTTCTACTCTTGTAGATGTTGGCCTTCCGATGGCTTCCCTAATTTCTACTCTTGTAGATGTCAAGCTCTGTTTGTGAAACTGTTTTTGCG	TTTG	TTTA	
pg1_Rv	aaacCGCAAAAACTCCTCCCGCATGGTCTTGGTACATCTACAAGAGTAGAAATTGCTTAGCCCAGAGAAAATGTAGAATCTACAAGAGTAGAAATTAC	TTTA	TTTA	
pg2_Rv	aaacCGCAAAAAATTGGCAGAGGGGCAGCGGGCTTATCTACAAGAGTAGAAATTTATGGGTATGATGAACGGAATTCATCTACAAGAGTAGAAATTAC	TTTA	TTTG	
pg2_Rv	aaacCGCAAAAAAGGGAAGCCATCGGAAGGCCAACATCTACAAGAGTAGAAATTATAAGGGCCAACTCACAGTAGAAATCTACAAGAGTAGAAATTAC	TTTA	TTTG	
pg4_Rv	aaacCGCAAAAACTCACAGGATACAGGCAGGATCCATCTACAAGAGTAGAAATTATTATCCGTTTACACGTGTGTCTATCTACAAGAGTAGAAATTAC	TTTA	TTTA	
pg5_Rv	aaacCGCAAAAACAGTTTCACAAACAGAGCTTGACATCTACAAGAGTAGAAATTAGGGAAGCCATCGGAAGGCCAACATCTACAAGAGTAGAAATTAC	TTTG	TTTA	
pg1_Fw	caccGTAATTTCTACTCTTGTAGATCATATGTCATTGCAGGGTGGAATAATTTCTACTCTTGTAGATGCACCAAGGCCATGTGGGAGGAATTTTTGCG	TTTA	TTTA	GS6
pg2_Fw	caccGTAATTTCTACTCTTGTAGATTAGTCCCAGTGCATCTCATGGGAAATTTCTACTCTTGTAGATACTGGAGGACTGCCTCTCACACTTTTTTGCG	TTTA	TTTA	
pg3_Fw	caccGTAATTTCTACTCTTGTAGATGGAACTGGTTCAGAACAGACCCAAATTTCTACTCTTGTAGATTACCTTATCTGCTTCCAGCTGACTTTTTGCG	TTTA	TTTA	
pg4_Fw	caccGTAATTTCTACTCTTGTAGATAGGTGCAACTGTAAACGGATAATAATTTCTACTCTTGTAGATCATGCCCACTCTGCAGGAGCAGATTTTTGCG	TTTA	TTTC	
pg5_Fw	caccGTAATTTCTACTCTTGTAGATATGGTAGCTTTCAGTCTGAGGGCAATTTCTACTCTTGTAGATAGGCCTTGGGCCATTTTGTAGGATTTTTGCG	TTTG	TTTG	
pg6_Fw	caccGTAATTTCTACTCTTGTAGATAGTATCTCAGCCCTGTTGCCATGAATTTCTACTCTTGTAGATCAGATGGGACCAACCCAGTGAAATTTTTGCG	TTTC	TTTG	
pg1_Rv	aaacCGCAAAAATTCCTCCCACATGGCCTTGGTGCATCTACAAGAGTAGAAATTATTCCACCCTGCAATGACATATGATCTACAAGAGTAGAAATTAC	TTTA	TTTA	
pg2_Rv	aaacCGCAAAAAAGTGTGAGAGGCAGTCCTCCAGTATCTACAAGAGTAGAAATTTCCCATGAGATGCACTGGGACTAATCTACAAGAGTAGAAATTAC	TTTA	TTTA	
pg2_Rv	aaacCGCAAAAAGTCAGCTGGAAGCAGATAAGGTAATCTACAAGAGTAGAAATTTGGGTCTGTTCTGAACCAGTTCCATCTACAAGAGTAGAAATTAC	TTTA	TTTA	
pg4_Rv	aaacCGCAAAAATCTGCTCCTGCAGAGTGGGCATGATCTACAAGAGTAGAAATTATTATCCGTTTACAGTTGCACCTATCTACAAGAGTAGAAATTAC	TTTA	TTTC	
pg5_Rv	aaacCGCAAAAATCCTACAAAATGGCCCAAGGCCTATCTACAAGAGTAGAAATTGCCCTCAGACTGAAAGCTACCATATCTACAAGAGTAGAAATTAC	TTTG	TTTG	
pg6_Rv	aaacCGCAAAAATTTCACTGGGTTGGTCCCATCTGATCTACAAGAGTAGAAATTCATGGCAACAGGGCTGAGATACTATCTACAAGAGTAGAAATTAC	TTTC	TTTG	

Color code: cloning overhangs, scaffold 1, sgRNA1, scaffold 2, sgRNA2, Poly(T)

Supplementary Table S5. Primers for deletion PCR including further specifications.

Name	Sequence (5'-3')	Annealing temperature (°C)	Expected amplicon size (bp)	Detection	Target
D_GS5_Fw	GCAAGCTGGAAATGCTTCC	58	1745	pg2	GS5
D_GS5_Rv	CAAAAGGCCTGCTTTAGTGAC				
D_GS5_2_Fw	GTGGCTTGTTAGTCTTGCCC				
D_GS5_2_Rv	GTGGAACTAGGAATTGCCAAGAC	58	837 and 1096	pg1 and pg3	
D_GS1_Fw	TAGGGTTGGGTCATGTGC				GS1
D_GS1_Rv	AGAAGAACGGGATTGTGGG	62	1729, 1272 and 1638	pg1, 2 and 3	
D_GS1_2_Fw	CCTGAGTGGAATTTTGATGGC				
D_GS1_2_Rv	GAAAATGTAAGCTGTTCATGCTG	52	690 and 550	pg4 and 5	
D_GS6_1_Fw	GCTTCTTGCTCTGAGGTGAG				GS6
D_GS6_1_Rv	CCCCCATCAGTAGCAATGTTC	58	1433	pg1	
D_GS6_2_Fw	GCTTCTTGCTCTGAGGTGAG				
D_GS6_2_Rv	GGGACATGTGCATTTATTTGATGC	58	1417 and 1260	pg2 and 3	
D_GS6_3_Fw	GTAGAGCAGAGAGTCTGAGCC				
D_GS6_3_Rv	GGAGCGGGAGTAATATGCTATC	52	2554, 667 and 560	pg4, 5 and 6	

Supplementary Table S6. Primers for non-deletion PCR including further specifications.

Name	Sequence (5'-3')	Annealing temperature (°C)	Expected amplicon size (bp)	Detection	Target
ND_GS5_Fw	CTTGGGTGGTTCTTGTGATG	60	902	pg1, 2 and 3	GS5
ND_GS5_Rv	CGCTTAAGTACGAATTACAGC				
ND_GS1_Fw	TACCTTTCAGTCTGAGGGCTC	60	185	pg1, 2 and 3	GS1
ND_GS1_Rv	GCTGGTTGCTCACCATGTC				
ND_GS1_2_Fw	GCCTATCGCAGGGATATCATG	56	591	pg4 and 5	
ND-GS1_2_Rv	GTCGCCAGTCTCATTGAGAAG				
ND_GS6_Fw	CCACTGGTCCAGGAAACTTG	60	647	pg1, 2 and 3	GS6
ND_GS6_Rv	GCATTGCAGCTGGTTAGTCAG				
ND_GS6_2_Fw	CTATGGCAGGGATATCATGGAG				
ND_GS6_2_Rv	AAGGTTGCTATAACCTCGAAGTCT	56	205	pg4, 5 and 6	

References

1 Pichler, J., Galosy, S., Mott, J. & Borth, N. Selection of CHO host cell subclones with increased specific antibody production rates by repeated cycles of transient transfection and cell sorting. *Biotechnol. Bioeng.* **108**, 386-394, doi:https://doi.org10.1002/bit.22946 (2011).

2 Schmieder, V. *et al.* Enhanced genome editing tools for multi-gene deletion knock-out approaches using paired CRISPR sgRNAs in CHO Cells. *Biotechnol. J.* **13**, e1700211, doi:https://doi.org10.1002/biot.201700211 (2018).
